# Bone histology for forensic anthropology: a technical review on the advances in microstructural analysis of taphonomically altered buried or subaerially exposed bone

**DOI:** 10.1007/s00414-025-03536-9

**Published:** 2025-07-10

**Authors:** Iris Sluis, Wilma Duijst, Tristan Krap

**Affiliations:** Faculty of Law and Criminology, Maastricht University, Minderbroedersberg 4-6, 6211 LK Maastricht, The Netherlands

**Keywords:** Bone histology, Bone diagenesis, Postmortem interval, Burial interval, Degree of preservation, Histological staining methods

## Abstract

Microscopy can be used to assess post-mortem bone degradation at a microstructural level. The duration of exposure to the environment may correlate to microstructural changes of the bone, and therefore, provide information about the post-mortem interval (PMI) or burial interval (BI). Through histological examination of thin bone sections, post-mortem microstructural changes can be investigated. The aim of this review is to provide an overview of histological staining methods that are applicable for studying microstructural taphonomical changes within bone, as well as reviewing and discussing the outcomes obtained through the application of these staining techniques in taphonomic research. In total, 45 stains were classified as promising to stain histological features that can undergo taphonomic changes in bone, including collagen and osteocytes. Examples of these stains include Mallory Trichrome staining, Gomori’s Trichrome staining, and Weigert’s resorcin-fuchsin method. The availability of protocols for use of the 45 stains on undecalcified, embedded and non-embedded bone sections was investigated. Protocols were available for 8 of the 45 stains on embedded bone sections, and for 2 of the 45 stains on non-embedded bone sections. A follow-up systematic literature search was conducted to obtain a comprehensive overview of the histological staining techniques that already have been tested within taphonomic research. Based on the outcome of the systematic literature search, only 3 out of the 45 different stains were tested, namely the Sirius Red/Fast Green staining, Haematoxylin–Eosin staining and Picrosirius red staining. These studies demonstrated that Sirius Red/Fast Green and Picrosirius red staining revealed significant changes in collagen degradation over time, useful for estimating the PMI or BI, while Hematoxylin–Eosin staining effectively portrayed bone morphology and its relation to a bone integrity index. Interestingly, 42 staining techniques have not yet been tested at all, leaving a huge knowledge gap. Therefore, the next step will involve testing the remaining stains on fresh and taphonomically altered, undecalcified bones, subsequently developing and validating a method to potentially relate the extent of degradation to the post-mortem interval or burial interval.

**Supplementary Information:**

The online version contains supplementary material available at 10.1007/s00414-025-03536-9.

## Introduction

After death, the body decomposes and after some time, the skeletal remains become exposed to the environment. Even at this stage, decomposition of bone continues, for example due to bacterial degradation. Post-mortem microstructural changes could correlate with the duration of exposure to the environment and thus provide information on the post-mortem interval (PMI), burial interval (BI), or taphonomic context [1, 2]. Bone histology can be used to assess post-mortem degradation through histological examination of bone sections [1, 3].

The decomposition of the cadaver is the first taphonomic agent altering bone quality. Decomposition fluids and enteric gut bacteria present during the active and advanced stages of decomposition negatively influence bone quality [4]. The process of degradation that occurs in bone is also known as bone diagenesis. It refers to a natural process encompassing both chemical and physical changes in the composition of the bone. These changes involve alterations in the proportions of organic (collagen) and inorganic components (hydroxyapatite, calcium, magnesium) when the bone is exposed to the environment [5].

The environment can be divided into different taphonomic spheres, which can be referred to as atmosphere, lithosphere, biosphere, hydrosphere, and cryosphere [6]. The atmosphere can be defined as a layer of gases, primarily composed of nitrogen, oxygen, argon, carbon dioxide, and other elements such as hydrogen, helium, and noble gases, that surrounds the Earth. The lithosphere is the outer, solid layer of the Earth. The biosphere includes all habitable regions on Earth, the hydrosphere is the layer of water on or near the Earth's surface, including seas, oceans, groundwater and water vapour, and the cryosphere encompasses frozen water parts [6]. The direct taphonomic context, in which the bone is located, has a strong influence on the speed of alterations it undergoes, as the (micro)environmental factors in a certain context can differ greatly [3]. Therefore, the diagenetic changes in the bone correlate with the environmental conditions [3, 7]. Paleontologist Efremov stated that what is not embedded is not preserved. He emphasized that the transition from the biosphere to the lithosphere is crucial, a process that occurs as a result of interlaced geological and biological phenomena [8]. Therefore, for this review, the focus is on the atmosphere, lithosphere, and biosphere.

Post active-decomposition environmental factors further influence bone diagenesis. Those environmental factors include pH, levels of exchangeable calcium and phosphorus, redox properties of the soil, ambient temperature, humidity, precipitation, sunshine hours, environmental soil compaction, microorganisms and bacteria [9, 10]. Temperature, particularly, has a significant impact on biological activities and biochemical reactions [11–15]. With increasing temperature, chemical reactions increase exponentially according to the Arrhenius equation, which describes how reaction rates accelerate with increasing temperatures. A 10 °C rise doubles reaction rates, including microbiological activity, however, bacteria also have an optimum range in which they grow [3, 16, 17]. Besides temperature, humidity also plays a major role, affecting bone mass loss [18]. Bacteria break down the bone through the excretion of acidic metabolites that affect the bone structure and lead to tunnel formation [3, 9, 10, 19]. There are various types of tunnelling, as described by Hackett (1981), such as Wedl tunnelling (fungal), linear longitudinal tunnelling (bacterial), budded tunnelling (bacterial), and lamellate tunnelling (bacterial) [3, 19]. These external factors vary across and within different spheres, influencing the degree of bone preservation and quality. For example, microorganisms are present in most soil types, but their quantity will vary depending on soil type, soil condition (i.e. physical, chemical and biological properties of the soil), and effective bone exposure. This can result in variability in diagenesis across the different environments [3, 7]. To assess the degree of diagenesis, various measurable parameters can be utilized according to Hedges (2002). These include collagen content, histological integrity, porosities such as water uptake potential, and crystallinity. Crystallinity is measured by the infra-red splitting factor, which involves properties related to crystal structure, density of defects, or crystal size and shape, and thereby helps in understanding diagenetic processes and bone preservation [20, 21]. Although these parameters generally increase in a correlated way, indicating a higher degree of diagenesis, their specific relationships vary depending on site-specific conditions and environmental factors, such as microbial activity and water movement [21, 22]. However, a more thorough understanding of the effects of environmental factors would greatly aid in gaining an underlying understanding of how most bone changes occur [21, 22].

Histological features can provide valuable information on post-mortem microstructural changes in bone that could potentially be linked to a degree of preservation [2]. The period of burial is inversely correlated with the bone integrity index [3], thus it provides information about the degree of bone preservation within the specific burial context. This preservation is important, as better-preserved bones, for example, retain more collagen (organic component) [2]. As the degree of preservation can indicate the duration of exposure, it can potentially be linked to a PMI or BI. A possible method for this is to stain the bone matrix to assess post-mortem diagenesis, *i.e.* histochemistry [3].

In histochemistry, a distinction is often made between staining decalcified and non-decalcified histological samples. Concerning decalcified samples, minerals such as calcium salts are removed from the bone biopt by means of acids or chelating agents (such as EDTA), after which the staining technique is applied. This way, only the organic component is available for staining [23]. However, undecalcified bone sections allow for the examination of the ratio between organic and inorganic components. For example, a relationship between collagenous and non-collagenous proteins remaining after a certain period of degradation. This collagen ratio can be determined by measuring the absorbance values of the Co- and NCo-specific dyes and comparing these values with known concentrations using standard curves [9]. Additionally, the crystal structure can be assessed by using the Hedges index to evaluate which original anatomical structures can still be recognized [21].

Moreover, a distinction is often made between thick and thin bone sections. Thick bone sections are approximately 2–3 mm, which is too thick for letting light pass through, and therefore are not suitable for light microscopy. In contrast, thin bone sections range from 10 to 15 microns, allowing light to pass through, thus well suited for histological examination. They can be prepared either manually using Frost’s'Rapid Manual Method' [24, 25] or automatically with a microtome [26]. However, bones are more fragile in archaeological contexts or when highly affected by taphonomic processes. In such cases, the preservation of collagen is also crucial; if the collagen is degraded, there will not be an organic component to stain, and decalcification then has no value besides being destructive. Therefore, the bones generally cannot be decalcified for processing with a microtome, where the sample is often surrounded by an embedding medium, for instance paraffin, plastic or resin, to support the sample [24, 26, 27].

Because of the importance of the ratio between organic and inorganic components, and the potential fragility of taphonomically altered bones, this study focuses on undecalcified bone sections, both embedded and non-embedded. This review aims to provide an overview of histological staining methods that are applicable for studying microstructural taphonomical changes within bone, as well as reviewing and discussing the outcomes obtained through the application of these staining techniques in taphonomic research. Therefore, we created an overview of histological staining methods applicable to bone, indicating what each dye stains and its intended purpose. For each staining method, we also assessed whether a protocol exists for use on thin undecalcified bone sections, both embedded and non-embedded. This overview was developed through an extensive literature search using Google, Google Scholar, and textbooks; As to the best of our knowledge, no review article was available yet. Subsequently, a systematic literature search was conducted to review whether these staining techniques have previously been applied to study the degree of preservation on bone sections or were related to the PMI or BI.

## The bone matrix and taphonomic agents

Bone consists of two types of structures, approximately 20% cancellous, spongy or trabecular bone and 80% cortical or compact bone. Although the structures have different morphological characteristics, both consist of the same chemical composition; an organic part (25%), an inorganic part (60–70%), and water (9.7%). The functions of bone tissue primarily involve providing support to the body, storing minerals and lipids, producing blood, protecting soft tissues and organs, and leverage [28]. Bone is an organo-mineral composite, wherein each component undergoes changes during diagenesis [18]. The organic phase consists mainly of collagen type 1, while the mineral phase includes hydroxyapatite crystals [18, 28].

Two processes are involved in the postmortem biological breakdown of tissues, namely autolysis and putrefaction. Autolysis involves the self-destruction of bone cells by enzymes, including proteases and DNases, which accelerate the destruction of tissue and component cells [29]. However, bone extracellular matrix proteins, such as collagen, are not susceptible to autolysis, according to Child 1995 [29]. As autolysis progresses, these enzymes break down tissues, creating a favourable environment for microbial activity [29]. Putrefaction, characterized by microbial breakdown by endogenous bacteria, further accelerates the decomposition process of the bones. The acidic metabolites generated during the putrefaction of the soft tissues during decomposition partially dissolve the bone matrix [30]. This creates enough space between the crystallites to allow penetration of the metabolites [29, 30]. Due to the increased porosity of the matrix, microorganisms can penetrate the surface and dissolve the bone from within. The bone matrix is broken down through hydrolysis, a process accelerated by enzymes secreted by microorganisms, known as collagenases. These enzymes are most efficient in an alkaline or neutral environment [30]. However, even apart from microbial activity, chemical (non-enzymatical) hydrolysis of collagen can also occur, due to factors such as the acidic environment of the soil. Although, this process occurs much slower than microbial enzymatic hydrolysis [10, 30]. Diagenesis results in loss of integrity of the bone matrix, osteocytes and endothelial cells, which often leads to channels and tunnels formation, caused by microorganisms or the environment. Structural changes also occur in the bone, which lead to the alteration in both the organic fraction of the bone matrix, and causing changes in the mineral component (bioapatite). Additionally, vascular spaces are filled with minerals present in the taphonomic context; a process called mineralization [3, 7].

However, in controlled laboratory experiments, the long-term diagenetic breakdown of bones described above does not apply. These experiments focus more on the effects of short-term exposure to various chemical conditions. In a highly acidic environment, such as hydrochloric acid (HCl), a bone can completely dissolve within 23 h. The histological structure can be affected after 19 h in 10% HCl, and, in 37% HCl, it fully degrades after 3 h [31–33]. However, as cortical bone is more resistant than spongy bone, time before complete dissolution in acid can vary [31]. According to Hartnett et al. (2011), sulfuric acid can dissolve the bone within 7 days, with the bone losing its integrity in just 1 day. Lye can dissolve the content of the marrow cavity but does not alter the structure or colour of the bone [32]. This indicates that, in acidic environments, the mineral components of bones are affected, as occurring during decalcification, while basic environments primarily impact the contents of the marrow cavity rather than the mineral components [31, 32, 34].

 In summary, the taphonomic agents affecting bones are of microbiological origin, including bacteria and fungi, and of chemical origin, including hydrolysis and mineralization. In both instances, chemical reactions form the basis of the changes in the bone, as it initiates the degradation of bone tissue, which is influenced by the environmental conditions the bones are in [10, 29, 30].

## Overview of staining techniques

An overview of histological staining techniques was created (see Table 1). The features that can be stained in bone are osteocytes (including nuclei), the bone matrix and its constituents (mostly collagen type 1), and external taphonomic agents (such as bacteria). These characteristics are highlighted in bold in Table 1. The focus lies on staining the organic components, as these degrade over time and are therefore potentially suitable for correlating with the time of degradation [2, 3]. Other stains that were not suitable for this purpose are shown in the Supplementary Material (see Supplementary Material S1), as these do not effectively highlight the organic components, or they may be better suited for staining mineralized structures rather than identifying organic degradation over time. This study also investigated whether staining protocols are available for the relevant staining techniques listed in Table 1, specifically for thin undecalcified bone sections, embedded or non-embedded. When these protocols were available, they were included in the Supplementary Material (see Supplementary Material S2). The overview was created by using combinations of search terms such as 'histological stains', 'histological staining techniques', 'staining bone', and ‘staining components of bone’. To access the availability of protocols, the name of each identified staining technique was combined with the terms 'undecalcified', 'embedded’ and ‘non-embedded'.

**Table 1:** Overview of different staining techniques. The table shows the staining, staining colour, application, if the protocol is available, and the sources. The features that can be stained in the bone are highlighted in bold

Staining	Staining colour	Application		Protocol	Sources
Hematoxylin–eosin staining (H&E staining)	Purple/blue **- Osteocytes:** Basophilic structures such as DNA in cell nuclei, RNA in ribosomes and rough endoplasmic reticulum Pink/Red **- Bone matrix:** Cellular and extracellular proteins such as cytoplasm and collagen fibres	Routine histopathological staining. Eosin is bound by the majority of structures in any tissue		Yes, embedded and non-embedded	[35–37]
Papanicolaou staining (PAP staining)	Blue **- Osteocyte:** Nucleus, **External taphonomic agents:** bacteria Red **-** Cytoplasm and keratin, mucus Yellow **-** Mucus in acidic environment Green **- Bone matrix:** Collagen	Routine cytopathological staining: cervical carcinoma, dysplasia		No	[35, 36]
Giemsa staining	Blue **-** Intracellular: **Osteocytes:** Nucleus and other basophilic substrates, **External taphonomic agents:** Bacteria Red **-** Intracellular: eosinophilic cytoplasm, mast cell granules. Basal membrane, parasites, **Bone matrix:** Collagen fibres Green **-** Melanin	Routine cytopathological staining: Differentiation between blood components Parasites (e.g., Trypanosoma, Plasmodium, Leishmania), Bacteria (e.g., Chlamydia, Borrelia, Helicobacter pylori, Rickettsia)		No	[35, 36]
Van Gieson stain	Red **- Bone matrix:** Connective tissue, collagen Black **-** Elastic fibres, **Osteocyte:** Nucleus Yellow **-** Muscles, cytoplasm	Detection of changes in connective tissue		No	[35]
Masson’s Trichrome staining	Blue **- Bone matrix:** Collagen fibres Red/pink **-** Muscle fibres and erythrocytes, cells and keratin, cytoplasm	Can identify cardiac fibrosis, pulmonary fibrosis, chronic kidney disease, and muscular dystrophy		No	[37]
Mallory Trichrome stain	Red **-** Muscle cells, **Osteocyte:** nuclei Blue **- Bone matrix:** Collagen Orange **-** Erythrocytes	Differentiates between collagen and muscle fibres		No	[38]
Heidenhain trichromes stain	Red **- Osteocyte:** Nuclei Orange **-** Muscle Blue **- Bone matrix:** Collagen Variety of colours **-** Cell cytoplasm	Differentiates between collagen and muscle fibres		No	[39–41]
Picrosirius red (PS) staining	Red **- Bone matrix:** Collagen type I and III, specific to amino acids lysine and hydroxylysine	To demonstrate the collagen fibres		No	[42]
Sirius Red/Fast Green	Red **- Bone matrix:** Collagen type I to V Green - Non-collagenous proteins (such as cells of muscle fibres)	Detecting all types and species of animal and human collagen		Yes, embedded and non-embedded	[9, 10]
Gomori’s trichrome	Black **- Osteocyte:** Nuclei Red **-** Cytoplasm, keratin, and muscle fibres Green or blue -**Bone matrix:** Collagen and mucus	Identify an increase in collagen fibres in the connective tissue or to differentiate between collagen and smooth muscle fibres		No	[43]
Azan trichrome stain	Red **-** Erythrocytes, **Osteocyte:** Nuclei Orange **-** Muscle Reddish **-** Glia fibrils Blue **-** Mucin Dark blue **-** Glomerular stroma, **Bone matrix:** Reticulum and collagen	Highlight collagen fibres within a tissue section		No	[38, 44]
Picrofuchsin stain	Red **- Bone matrix:** Collagenous fibres Yellow **-** Muscle tissue and glia fibrils Red to yellow **-** Amyloid, hyaline, colloid, and mucus	Used for staining collagenous connective tissue, muscle tissue, cornified epithelium, glia fibrils and cytoplasm		No	[45]
Lillie’s Trichrome	Black **- Osteocyte:** Nuclei Red **-** Cytoplasm and muscle Blue- green **- Bone matrix:** Collagen	Used for muscle and collagen		No	[46]
Miller’s Elastic Tissue stain	Black **-** Elastic fibres, **Osteocyte:** Nuclei Yellow **-** Cytoplasm Red **- Bone matrix:** Collagen	Demonstrate elastic fibres in tissue		No	[47]
Bile stain, Hall’s	Green **-** Bilirubin Pink to red **- Bone matrix:** Collagen Yellow **-** Background of muscle and cell cytoplasm	Pigments and minerals		No	[48, 49]
Movat’s Pentachrome	Black **-** Elastic fibres, **Osteocyte:** Nuclei Intense red **-** Fibrin Red **-** Muscle Yellow **- Bone matrix:** Collagen and reticular fibres Blue **-** Ground substance muccin	Highlights fibrin, mucin, and collagen in a tissue section		No	[44, 50]
Colloidal Iron stain	Blue - Acid mucopolysaccharides Red **- Bone matrix:** Collagen **Osteocyte:** Nuclei	Stain acid mucosubstances		No	[51, 52]
Weigert’s resorcin-fuchsin method	Blue-black - Elastic fibers Light blue-black - **Osteocyte**: Nuclei Pink or red **- Bone matrix:** Collagen Yellow - Other tissue	Colour elastic fibers		No	[38, 51, 53]
Fraser Lendrum stain	Red **-** Fibrin, keratin and some cytoplasmic granules Orange **-** Erythrocytes Green **- Bone matrix:** Collagen	Fibrin		No	[51, 54]
Oxone-Aldehyde- Fuchsin-Halmi stain	Deep violet **-** Oxytalan fibres Lighter purple **-** Keratin, tooth pulp, mast cells, and basement membranes Blueish-brown **- Osteocyte:** Nuclei Green **-** Epithelial cells Green- greenish yellow **- Bone matrix:** Collagen	To primarily visualize oxytalan fibres and connective tissue within periodontal tissue		No	[44]
Combined Massons Elastin	Black **-** Elastic fibres, **Osteocyte:** Nuclei Green **-** Mucin, **Bone matrix:** Collagen Red **-** Keratin, cytoplasm, and muscle fibres	Highlight connective tissue fibres within a tissue sample		No	[44]
Cason’s trichrome	Red **-** Cytoplasm, **Osteocyte:** Nuclei Blue **- Bone matrix:** Collagen Orange **-** Erythrocytes	Differentiate collagen		No	[38]
Weigert’s Elastic tissue	Purple black **-** Elastic fibres Red **- Bone matrix:** Collagen Yellow **-** Cytoplasm Blue **- Osteocyte:** Nuclei	For elastic fibres		No	[39, 55]
Picrofuchsin Van Gieson	Black to brown **- Osteocyte:** Nuclei Red **- Bone matrix:** Collagen Yellow **-** Muscle and glia fibrils Yellow to red **-** Colloid, mucus, hyaline, amyloid, cornified epithelium	Differentiate collagen from connective tissue		No	[56]
Methylene blue	Blue **- Osteocyte:** Nucleus	Good to differentiate between DNA and RNA in tissues		No	[37, 57]
Methylene Green	Blue-green to green **- Osteocyte:** DNA Pink to red **- Osteocyte:** RNA Pink **-** Mast cells granules	To identify DNA, RNA and Mast Cell Granules		No	[37, 58]
Methyl Green Pyronin	Pale green **- Osteocyte:** DNA Pink red **- Osteocyte**: RNA	DNA and RNA can be detected		Yes, embedded	[59]
Toluidine Blue	Deep blue **- Osteocyte**: Nucleus	It will also stain polysaccharides a pink/red color (metachromasia)		Yes, embedded	[37, 60]
Gram stain	Purple **- External taphonomic agents:** Gram-positive bacteria; contain a thick layer of peptidoglycan Pink **-** External taphonomic agents: Gram-negative bacteria; have a thin layer of peptidoglycan and more lipids in the cell	Differentiating bacterial species		No	[35]
Pappenheim staining (MGG staining)	Blue **- Osteocytes:** Nucleus, other basophilic substrates, basophilic granules Red **-** Erythrocytes, eosinophilic granules	Differentiation between blood and bone marrow components		No	[35]
Masson–Goldner staining	Red **-** Cytoplasm, erythrocytes, fibrin, muscles, **Osteocyte:** osteoid Green **-** Mucus, **Osteocytes**: Bones, connective tissue	Detection of changes in connective tissue		Yes, embedded	[35]
Periodic acid-Schiff reaction (PAS reaction)	Blue **- Osteocyte:** Nucleus Red **-** Neutral glycosaminoglycans, mucopolysaccharides, carbohydrates, glycogen	Fungi (e.g., aspergillosis), parasites, macrophage staining in Whipple disease, glycogen storage diseases, alpha 1 antitrypsin deficiency		Yes, embedded	[35, 36]
Prussian blue reaction	Blue **-** Iron in mitochondria Red **-** **Osteocyte:** Nucleus	Iron detection; siderosis (iron overload), alveolar macrophages		No	[35, 36]
Congo Red	Blue **- Osteocyte:** Nucleus Red **-** Amyloid (β-fibrils, green after polarization)	Amyloidosis, amyloid deposits		No	[35, 36]
Von Kossa staining	Red **- Osteocyte:** Nucleus Black **-** Calcium phosphate Pink **-** Cytoplasm	Calcification		Yes, embedded	[35, 44]
Safranin O	Orange to red **-** Cartilage and mucin Black **- Osteocyte:** Nuclei Green **-** Background	Detection of cartilage, mucins and mast cells on formalin fixed, paraffin embedded tissue, or frozen sections		Yes, embedded	[44, 50]
Crystal Violet	Violet **- Osteocyte**: DNA in the nucleus of a cell	Can stain glia and neurons		No	[61, 62]
May-Grünwald-Giemsa stain	Blue **-** Cytoplasm of lymphocytes and monocytes and cytoplasm proteins, **Osteocyte:** Nuclei (DNA and RNA/chromatin structure), basophilic granulations Red orange **-** Red blood cells (haemoglobin), basophilic and eosinophilic granulations and neutrophilic cytoplasm proteins Purple **-** Azurophilic granulations of the cytoplasm **Osteocyte:** Nuclei (DNA and chromatin structure)		Used in hematology to differentiate and count different blood cell population in cellular preparations (cytology)	No	[63, 64]
Verhoeff’s van Gieson stain	Gray to black **- Osteocyte:** Nuclei Black **-** Elastic fibres		Highlights network of elastic fibres	No	[43, 49]
Trommsdorff’s Malachite Green	Green in red rods or debris **- External taphonomic agents:** spores		Bacterial spore staining	No	[65]
Azocarmine stain	Deep red **- Osteocyte:** Nuclei Pale red **-** Cytoplasm		To stain acidic cellular components, such as nucleic acids or acidic proteins	No	[39, 66]
Phloxine B stain	Blue **- Osteocyte:** Cell nuclei Pink **-** Acidic mucosubstances		Used for human-medical cell diagnosis	No	[67]
Copper stain	Light brown to red **-** Copper deposits Blue **- Osteocyte:** Nuceli		Demonstration of copper deposits in tissue sections	No	[51]
Gridley’s stain	Purple **- External taphonomic agents:** Fungi Yellow **-** Background		For fungi	No	[51, 68]
Fluorescent antibody stain	Red **-** Tubulin, mitochondria, endoplasmic reticulum Green **-** Actin, endoplasmic reticulum Blue **- Osteocyte:** Nucleus		Bacteria (e.g., Treponema pallidum, Pneumocystis jirovecii), Protozoa (e.g., Giardia, Cryptosporidium)	No	[35]

## Relevant studies for estimating the PMI based on histology

A total of 45 staining methods are available for staining collagen and osteocytes in bone, (see Table 1). The question rose whether these can be effectively used to study bone preservation and subsequently estimate the PMI. As this review aimed to obtain a comprehensive overview of the studies that have attempted to relate bone degradation to a PMI or BI using histological staining techniques, a systematic literature review was conducted. Electronic databases were used for this systematic literature search, namely PubMed and ScienceDirect. The recording year limit was from 1980 to 2023 (the search included all articles until July 2023), and the language of the articles was limited to English. The 1980 cut-off was set to ensure the focus was on more recent methodologies and techniques. The keywords combinations used were the following; ‘bone histology’, ‘forensic anthropology’, ‘staining techniques’, ‘collagen degradation’, ‘histology’, ‘postmortem interval’, ‘current state of study histology’. Articles were only included if they contained buried or subaerial decomposition; articles about decomposition in aquatic environments were excluded by title review. Besides, studies related to PMI estimation, histological staining techniques, bone diagenesis, collagen degradation, and osteocyte degradation were included. Articles were initially selected based on title, followed by abstract review, then the full text was read by the first author (I.S.). In cases of uncertainty regarding inclusion, the second (W.D.) and third (T.K.) authors reviewed the articles to make a final decision. Using the 'snowball method',the bibliographies of articles included in the abstract were used to gather additional literature, which was then selected in the same way as described above [69]. In total, there were 2810 hits based on the search terms, see Table 2. After title inclusion, 115 articles remained and following abstract inclusion, 77 articles were included in the review. After full-text assessment, 12 articles met all criteria. Using the snowball method, 4 additional articles were examined, of which 1 was included. This resulted in a total of 13 articles that were selected for the final analysis.

The following information was extracted out of the final 13 articles: the population/sample type/sample size/PMI, the goal, sample grouping and setting, the method and the results, resulting in the overview shown in Table 3. In these studies, three studies utilized Sirius Red/Fast Green staining [9, 10, 70], two studies employed Hematoxylin-eosin (HE) staining [3, 42], and Picrosirius Red staining was applied once [42]. In all studies, embedded bone was used. In the study by Delannoy et al. (2018) and Astolphi et al. (2019), the bones were also decalcified [3, 42]. In the studies by Boaks et al. (2014), Jellinghaus et al. (2018) and Jellinghaus et al. (2019), undecalcified bone was used [8, 9, 70]. The remaining studies primarily focused on other methods to detect post-mortem changes, such as UV fluorescence, infrared, and spectrophotometric methods [2, 71–77]. These results are highlighted in bold in the Table 3.

**Table 2:** Systematic literature search. The table shows the search terms, hits, title inclusion and abstraction inclusion

Search term	Hits	Title inclusion	Abstract inclusion	Article inclusion (indicated in parentheses when the article was not yet included)
**PubMed**				
Bone histology forensic anthropology	11	4	3	1 (1)
Bone histology bone matrix postmortem interval	7	1	1	0
Human cortical bone histology stain	309	6	5	2 (1)
Staining of bone taphonomy histology	45	6	3	1
Bone histology forensic taphonomy	186	16	8	2 (1)
Postmortem interval estimation bone histology	46	15	13	6 (6)
**ScienceDirect**				
Bone histological stains forensic anthropology	109	14	8	4 (1)
Collagen degradation at a histological level forensic anthropology	58	14	8	4 (1)
Current state of study bone histological staining techniques forensic anthropology	45	8	4	2
Bone histology bone matrix postmortem interval	1072	7	4	2
Histological stains bone matrix post mortem interval	730	7	5	2
Overview histological staining techniques anthropology	51	2	2	0
Staining of bone taphonomic histology	129	13	9	2
Histochemical approach bones taphonomy	9	2	1	1 (1)
**‘Snowball effect’**	4			1 (1)
**Total**	**2810**	**115**	**77**	**13**

**Table 3:** The relevant studies that have already been conducted related to estimating the PMI based on skeletal remains using various staining techniques. The table shows the study, followed by the population/sample type/sample size/PMI, the goal, the sample grouping and setting, method and the results. Staining methods or detection techniques are highlighted in bold

Study	Population/ sample type/ and sample size (N)/PMI	Goal	Sample grouping and setting	Method	Results
Bell et al. 1996 [71]	*N*= 11 human skeletal specimens + teeth, Canada, PMI: 3 months – 83 years.	Assess post-mortem microstructural changes in bones.	Thick sections were made from bone and teeth, using a wet diamond-edged circular saw. After embedding, the sections were re-cut using Isomet-11-1180 circular saw.	The sections were **examined using a Cambridge Stereoscan S4-10 or** **Zeiss DSM 962 scanning electron microscope (SEM).**	Post mortem change was found at the earliest time investigated, 3 months after death. However, it is possible that changes occur earlier.
Jaggers and Rogers 2009 [72]	*N*=120 non-human bone samples (femur, tibia, and fibula) of Sus scrofa. PMI: 60 and 150 days.	Document morphological changes and establish PMI feasibility.	Group A: 60 samples. Group B: 60 samples. Both groups were buried at 2 different locations with the same weather conditions but different soil compositions. There were 30 graves per location, with 2 bones buried per grave.	After 60 and 150 days, the samples were excavated and **analysed for macroscopic changes such as colour, texture, amount of soft tissue, number of cracks, et**c. The collected data also included bone weight and hydration.	No significant changes observed in bone color, texture, or condition over 150 days. The study suggests that it will take at least a year and 5 months before significant morphological changes appear on the bone.
Ramsthaler et al. 2011 [73]	*N*= 39 human long bones, PMI = 0–2500 years. And *N*=5 non-human mammal bones (cow, horse, pig, and dog).	Investigating whether tests originally designed to detect blood can also be used to determine the PMI.	The bones were stored in dry conditions (up to 10 years, 16–18 °C). Cross-sections were made of the shaft.	**UV reflection** of a fresh cross-section and **luminol test** were applied, and additionally, two new methods were employed**: the Hexagon-OBTI® test and the Combur® test**.	UV reflection correlates with PMI (< 50 years); luminol positive for recent samples (< 10 years); Hexagon-OBTI® and Combur® not suitable for PMI estimation.
Hoke et al. 2013 [74]	*N*=165 Sus scrofa femur bones. Group 1: 40 bones buried in wooden crates. Group 2: 20 bones in plant material. Group 3: 90 bones in plastic containers. Group 4: 15 bones buried in the ground.	Assess organic staining for PMI estimation.	In Groups 1–3, femur epiphyses were removed using a reciprocating saw to extract marrow fat. Skeletal elements were then defleshed and degreased in an 80–20% dichloromethane-acetone mixture before immersion in tap water. Group 2 consisted of bones that had been mostly defleshed and previously buried at a decomposition site.	**Munsell Soil Color Charts and 40W daylight lamp** for color and staining documentation after 0, 6, and 12 months.	Organic staining visible within 2 months. Allow for determining the significance of remains and the time they have been exposed to organic materials.
Boaks et al. 2014 [9]	*N*=5 Sus scrofa (10 bones, femur and humerus) (and femur and tibia for the period of 2 months decomposition).	Detect collagen (Co-) and non-collagenous (NCo-) protein breakdown for PMI estimation.	200–300 mm cross-section, 2, 4, 6, 10, and 12 months of 10 juvenile long bones.	**Sirius Red/Fast Green staining**; measured at 540 nm and 605 nm.	A statistically significant change over time was observed. The method was not sensitive enough for PMI estimation but proved useful as a tool to determine the concentration of Co/NCo over time.
Kontopoulos et al. 2016 [2]	*N*=30 Sus scrofa carcasses (25 kg) were buried or placed on the ground surface. *PMI* = 6/7 years. And *N*= 3 small pig carcasses (2–3 kg), wrapped in clothing/carpet	The primary objective was to understand the extent to which microbial attacks affect bone preservation in the early diagenetic stages.	33 thin sections (50 μm) were prepared using a Leitz 1600 annular saw microtome.	Thin sections were examined under both **ordinary polarized and cross-polarized transmitted light** using a Leica microscope (Leitz Laborlux 12 Pol S) at magnifications ranging from ×25 to ×100.	Histological examination reveals significant variability between macroscopic and microscopic preservation, indicating that surface preservation and collagen loss are not consistent. Diagenetic changes in bone are complex and may exhibit different patterns, suggesting that each bone may follow a unique diagenetic trajectory due to variations in biomechanical characteristics.
Sterzik et al. 2016 [75]	*N* = 30 human femur bones, PMI = 1–49 years.	To more accurately determine the forensic-relevant PMI (< 50 years) in human skeletal remains.	Cross-sections were made using an oscillating autopsy saw (Buehler). One side of the was sanded with fine sandpaper (grit 1200) to prevent reflection artifacts. A forensic light source (Lumatec Superlite 410, 490 nm) and a UV lamp (365 nm and 254 nm) were used.	**The proportion of fluorescent surface (surface size, percentage of the surface) was examined**, and in addition, a test was conducted at 490 nm; a completely new, unpublished method.	Blue fluorescence indicates PMI < 50 years; red fluorescence more precise. The UV-induced fluorescence was not very useful in determining the PMI.
Sterzik et al. 2018 [76]	*N* = 20 human femur bones from 20, PMI ≥ 50 years.	Extend the previous study by Sterzik et al. (2016).	Similar method as in Sterzik et al. (2016).	**Similar as in Sterzik et al. (2016)** and the results of the previous study were combined with the results of the current research.	The presence of blue and red fluorescent surfaces > 1% can be considered as a marker to exclude a PMI ≥ 50 years. The presence of blue and red fluorescent surfaces < 1% indicates a PMI ≥ 50 years, but excluding a PMI < 50 years is not possible.
Delannoy et al. 2018 [42]	*N* = 12 human ribs (1st and 4th rib from right side) from 6 individuals.	Study collagen degradation and microbial impacts.	The ribs were placed in an outdoor environment (under cover, in clay) and sampled every 3 months. After 24 months, the ribs were extracted from the environment. Transverse sections (5 mm) were made with diamond disc.	The ribs were fixed in 10% neutral buffered formalin and after that, the bone fragments were decalcified. The sections were stained with **H&E staining** to study general morphology **and Picrosirius red (PS)** to demonstrate the collagen fibres observed under polarized light.	From 6 to 24 months postmortem, the bone lamellar architecture became disorganized. Within the first year postmortem, osteons displayed erosion around the Haversian canal. By 24 months postmortem, this degradation extended to the outer cortical bone, accompanied by further disorganization of the lamellar architecture. This collagen degradation was primarily caused by chemical hydrolysis, rather than bacterial activity.
Jellinghaus et al. 2018 (revised method of Boaks et al.) [10]	*N* = 16 left femur bones from Sus scrofa, *PMI* = 3 months.	Review Boaks et al. method for reproducibility and forensic use.	16 left femur bones from pigs were divided into 2 groups and stored in 4 containers filled with soil. Every month, a slide of a bone (0.7×2.5 cm in size, rectangular) was removed. The hole in the bone was filled with silicone grease. From each bone sample, a section of 250 micrometres was made.	**The Sirius Red/Fast Green Collagen Staining Kit from Chondrex** was used. Staining was eluted with extraction buffer and measured using Nanodrop at 540 nm and 605 nm.	The Co/NCo ratio can be used as a source of information for determining PMI. However, many steps are still needed to utilize collagen degradation as a PMI estimator.
Jellinghaus et al. 2019 [70]	*N* = 35 human femur bons, PMI = 3–5 years. And *N* =11 skeleton, *PMI* = 153 – 171 years.	Assess Boaks et al. method for human bones and compare Co/NCo ratio in humans and pigs.	All bone samples were defleshed, dried, and stored in open plastic bags in a dark place. Using an oscillating autopsy saw (Beuhler), a rectangular piece with a width of approximately 1 cm was cut out from the femurs. These pieces were then stored in dry and open plastic bags at room temperature.	The bone samples were stabilized in a resin base and cured. Sections were made into three thin sections with a width of 250 micrometers using a Leica SP1600 saw microtome. **The Sirius Red/Fast Green Collagen Staining Kit from Chondrex** was utilized. Staining was eluted with extraction buffer and measured using Nanodrop at 540 nm and 605 nm.	Co/NCo ratio decreases over time. Applicable in both pigs and human bones. The relationship between PMI and Co/NCo ratio decreases in males, while it increases for female individuals. The method can be used as initial advice for PMI estimations or to narrow down the PMI interval.
Astolphi et al. 2019 [3]	*N* = 39 human femur bones from males	Explore diagenesis in compact bone in tropical settings.	Cross-sections of 2 cm^2^ were obtained from the midshaft of the femur. The control group consisted of 2 cm^2^ cross-sections from the midshaft of the femur of 5 individuals after routine amputations.	The cleaned bone samples were fixed directly in 10% neutral buffered and the samples were decalcified. Sections of tissue paraffin blocks, 5 μm thick, were stained with **Hematoxylin-eosin (HE) staining.** Histological analysis was performed using light microscopy. Bone matrix loss was analysed using ImageJ.	Bone integrity and matrix loss increase with burial duration.
Caruso et al. 2021 [77]	*N* = 8 long bones (femur, tibia and humerus) of juveniles	Study diagenetic processes in juvenile bones.	Thin cross-sections were made and analysed using optical microscopy. Bone images were obtained using IScapture® software (version 3.6.7).	Thin cross-sections were made and analysed using **optical microscopy, SR-μCT, EMPA, FT-IR, XRPD.** The histological assessment was quantified using the Oxford Histological Index (OHI).	The organic fraction is highly variable in quality, quantity, and arrangement. Juvenile bones significantly differ from adult bones in terms of preservation and diagenetic changes, influenced by intrinsic characteristics (shape, porosity, and histological structures), and external factors (burial environment).

## Discussion

The aim of this review was to provide an overview of histological staining methods that can be used to study the microstructural changes within bone due to taphonomic agents, and review and discuss the outcomes obtained through the application of these staining techniques in taphonomic research. Multiple studies have been conducted on microstructural taphonomic changes within bone, collagen degradation, and relating degradation to a postmortem interval [2, 3, 9, 10, 42, 71–77]. However, it is noteworthy that, according to the results from this literature review, the only histochemical stains used in these studies are Hematoxylin–eosin (HE), Sirius Red/Fast Green staining and Picrosirius Red staining [3, 9, 10, 42, 70]. The overview of histological staining methods (Table 1) shows that many more staining methods have potential for highlighting microstructural taphonomic changes in bone when observed under the microscope.

A total of 45 stains were identified as promising for demonstrating collagen and osteocytes in bone tissue. For staining techniques targeting osteocytes, the ratio between the number of osteocyte nuclei and the number of osteocyte lacunae over time, also referred to as the bone integrity index, could be determined. This index may serve as an indicator of the degree of preservation and could be potentially linked to a PMI [3]. Examples of stains that may be of interest for this purpose include Heidenhain trichrome stain, Gomori’s trichrome, Toluidine Blue, and Azan trichrome stain. Hematoxylin-Eosin could also be suitable for this purpose, as it was used in experimental research on chondrocytes in taphonomically degraded hyaline cartilage [78]. Over a 28-day period, a decline in chondrocytes was observed at various time points [78]. Concerning staining techniques targeting the bone matrix, the focus will primarily be on determining the ratio between collagen and non-collagenous proteins [2, 9]. Picrosirius Red (PS) staining and Picrofuchsin stain, in addition to Sirius Red/Fast Green, are examples of stains that could be applied for this purpose. Sirius Red/Fast Green was already applied in the research by Boaks et al. (2014), Jellinghaus et al. (2018), and Jellinghaus et al. (2019), as well as Picrosirius Red in the study by Delannoy et al. (20218), where collagen degradation was observed in taphonomically altered remains [9, 10, 42, 70]. For staining techniques highlighting external taphonomic agents (such as bacteria), the emphasis is on demonstrating various types of tunneling as described by Hackett (1981), which could provide valuable information on post-mortem microstructural changes [2, 19]. Examples of stains that could be used for this purpose include Gram stain, Trommsdorff’s Malachite Green, and Gridley’s stain. The limited research on these stains in combination with taphonomically altered bone and PMI estimations highlights the need for further experimental work.

The studies using these stains on taphonomically altered remains showed that Sirius Red/Fast Green demonstrated a statistically significant change in collagen integrity over time [9, 10, 70]. The staining technique was successfully applied on both pig and human bones to obtain information about the PMI [9, 10, 70], and can be used as initial advice for PMI estimations or to narrow down the PMI interval, according to Jellinghaus et al. (2019). Although there is a promising method for assessing the collagen ratio in bone and relating it to known decomposition times, a model for estimating an unknown PMI based on the collagen ratio has not yet been developed. Such a model would also need to be context-specific, accounting for different soil types or taphonomic environments, as there is still insufficient knowledge about the influencing factors [9, 10, 70]. Picrosirius Red demonstrated a time-dependent change in collagen degradation [42]. Studies on the use of Hematoxylin–Eosin staining show that this staining is suitable for representing the general morphology of the bone and relating it to a bone integrity index, but not for PMI estimations so far [3, 42].

In the study by Astolphi et al. (2019) [3], an inverse correlation was found between the burial period and the bone integrity index. This index is based on the ratio of osteocyte nuclei to lacunae and reflects the degree of bone preservation [3]. Although the bone integrity index may be related to the PMI, there is currently no validated method to establish this relationship. Even though significant loss of the bone matrix was observed due to diagenesis, this was not correlated with the burial period. This is likely due to varying burial conditions, such as differences in soil chemistry and hydrology. The lack of a detailed excavation context may also have influenced the interpretation of diagenetic changes. Despite the lack of a clear relationship between burial period and bone matrix loss, the bone integrity index appears to be sensitive to burial conditions [3]. These findings, together with studies by Boaks et al. (2014) [9], Jellinghaus et al. (2018) [10] and Jellinghaus et al. (2019) [70] suggest that collagen and osteocyte degradation could be an indicator for estimating the PMI. A first step was also taken to relate collagen and osteocyte degradation to PMI, with promising initial results. However, the method needs to be tested and validated more extensively to develop a model to estimate an unknown PMI based on the collagen and osteocyte ratio in bones [3, 9, 10, 70].

It is known that many factors can influence bone diagenesis, such as external variables including temperature, the environment, or the immediate taphonomic context in which the bone is located, as the human body interacts with the burial environment [77]. Jaggers and Rogers (2009) [72] concluded in their study that knowledge of weather conditions for the time period of interest is necessary for making meaningful conclusions regarding PMI [72]. Schotmans et al. (2004) [79] concluded that not only soil versus gut bacteria, nor bioerosion solely related to post-mortem events, but that the broader depositional context should be analyzed on a case-by-case basis, in combination with a self-critical use of methods and interpretations [79]. Bone diagenesis is therefore a difficult process to predict and reconstruct, and a thorough understanding of the effects of environmental factors would greatly help in understanding how most bone changes occur [21, 22]. Having more information about the immediate taphonomic context in which the bones are found is important to better understand the conditions surrounding bone preservation. A holistic approach is crucial for estimating the PMI with acceptable accuracy. This invites further research into differences in bone preservation and various taphonomic environments. Information about the taphonomic environment in which the bone was located is also crucial for applying the specific staining technique. Various factors (such as soil composition, pH, temperature, and microbial activity) can affect the effectiveness and performance of the staining technique [80]. For instance, the crystal structure of bones may become acidified when exposed to acidic environments. Stains that perform better in alkaline conditions may yield distorted results or fail to stain at all if applied to this acidified bone. This could lead to misinterpretations, as specific characteristics may not be stained. Therefore, it would be important to investigate pretreatment, such as neutralization of the bone, before applying the staining technique [80, 81]. Understanding the taphonomic context is essential for making informed decisions about these procedures. However, much experimental work is still needed in this area to ensure the effectiveness and reliability of the staining technique, allowing for accurate interpretations to be made.

Since it has become clear that additional staining techniques may be suitable for detecting organic components in bone, an important first step in future research would be to test these stains on fresh, undecalcified thin bone sections to determine if they are indeed effective in staining collagen and osteocytes. This is relevant to investigate, as many studies in histochemistry focus on the decalcified bone sections. Since the calcium salts have been removed from these bones, they are easily processed mechanically into thin sections for histological analysis and stained for microscopic analysis [23, 82]. However, decalcification can lead to the loss of relevant minerals, such as those needed to assess the ratio of the organic component to the inorganic component, making it essential to test the stains on non-decalcified thin bone sections.

Protocols for non-embedded undecalcified bone sections are currently available for only 2 out of the 45 stains identified, and protocols for embedded undecalcified bone sections exist for 8 out of 45. This highlights a significant gap in research. Developing and testing protocols for the remaining stains on embedded and non-embedded undecalcified fresh bone sections is the crucial next step. The research should also focus on whether a combination of multiple staining techniques provides more accurate and reliable results compared to individual methods, and on identifying which staining methods offer the best contrast between stained collagen, osteocytes, and the background or other less relevant structures. Additionally, it is also important to test these stains on degraded, undecalcified thin bone sections to assess their suitability under different stages of degradation.

Another recommended next step would be to explore whether the collagen/non-collagen ratio and osteocyte ratio can be related to the degree of degradation and potentially the PMI. To achieve this, previously tested stains in other studies, such as the Hematoxylin–eosin (HE), Sirius Red/Fast Green, and Picrosirius Red should be further explored, while alternative stains, such as Van Gieson and Mallory Trichrome, may also offer value due to their high contrast between relevant structures. Additionally, developing and validating a method to estimate the PMI based on skeletal elements could offer significant forensic value, provided that such a method adheres to the quality standards required for admissibility in court [83–86].

Although histology combined with microscopy can provide valuable information, the method it is not without limitations. The assessment of microscopic images is mostly subjective, depending on the experience and interpretation of the examiner. In addition, different bone types (e.g. compact vs. trabecular bone) can respond differently to staining techniques, which can affect the intensity and contrast of the staining. In the study by Boaks et al. (2014) [9], it was found that the cavities in trabecular bone contained more inorganic materials that were stained, thereby affecting the ratio of collagen to non-collagenous proteins in the bone section [9]. This can all lead to variability in the interpretation of staining results. Histological images are often interpreted using the Hedges scoring index [21], or by assessing the presence or absence of specific histological features. As mentioned earlier, it is important to have information about the taphonomic context of the bone to determine whether and which pre-treatment steps are necessary for the appropriate application of the staining technique and interpretation of the results. To reduce the subjectivity and variability of staining results, multiple examiners could review the histological images. However, for future prospects, it is interesting to involve AI-based tools (Artificial Intelligence), such as Cellpose, in the analysis of histological preparations to recognize patterns. AI-based tools can identify abnormal structures or contribute to recognizing and calculating the ratio between organic and inorganic components [87, 88]. This can improve the efficiency of the analysis, as well as the consistency in finding new ratios and trends. In this way, human errors could be minimized, and patterns could be better recognized, leading to faster and more efficient procedures.

## Conclusion

In this review, an overview of histochemical staining methods to study microstructural taphonomic changes within bone was created. Additionally, a review of histological staining methods that have already been tested in taphonomic research now allows for more targeted investigation. This will help identify which histological staining methods are most suitable for demonstrating collagen and osteocyte degradation in undecalcified thin bone sections. Out of 45 potential histochemical stains identified for analysing bone degradation, only a few, such as Hematoxylin–eosin (HE), Sirius Red/Fast Green, and Picrosirius Red, have been tested thus far, with promising results for identifying collagen or osteocytes as an indicator for the decomposition time. However, these stains now need to be tested on both embedded and non-embedded undecalcified bone sections. Besides, staining protocols were only found in 2 out of the 45 stains for non-embedded undecalcified bone sections, and for 8 out of 45 for embedded undecalcified bone sections. The next step is to develop and test the remaining protocols for undecalcified fresh bone sections, especially focusing on whether combining multiple staining techniques could provide more accurate and reliable results and determining which staining methods offer the best contrast between relevant and non-relevant structures. Examples of stains that are interesting for the contrast they provide include Van Gieson stain, Mallory Trichrome stain, Heidenhain trichrome stain, and Copper stain. Further research could also focus on the already tested stains, namely Hematoxylin–eosin (HE), Sirius Red/Fast Green staining, and Picrosirius Red staining, as these stains have demonstrated the ability to reveal bone degradation by assessing collagen and osteocyte degradation. Ultimately, a method must be developed, tested under differing conditions, and validated to reliably estimate the postmortem or burial interval.

## Supplementary Information

 Below is the link to the electronic supplementary material.

ESM 1 (DOCX 53.0 KB)

## References

[1] Forbes SL (2008) Potential determinants of postmortem and postburial interval of buried remains. In: Soil analysis in forensic taphonomy (pp. 225–246) CRC Press.

[2] Kontopoulos I, Nystrom P, White L (2016) Experimental taphonomy: post-mortem microstructural modifications in Sus scrofa domesticus bone. Forensic Sci Int 266:320–328. 10.1016/j.forsciint.2016.06.024 27368073 10.1016/j.forsciint.2016.06.024

[3] Astolphi RD, de Seixas Alves MT, Evison MP, Francisco RA, Guimarães MA, Iwamura ESM (2019) The impact of burial period on compact bone microstructure: histological analysis of matrix loss and cell integrity in human bones exhumed from tropical soil. Forensic Sci Int 298:384–392. 10.1016/j.forsciint.2019.03.008 30928778 10.1016/j.forsciint.2019.03.008

[4] White L, Booth TJ (2014) The origin of bacteria responsible for bioerosion to the internal bone microstructure: results from experimentally-deposited pig carcasses. Forensic Sci Int 239:92–102. 10.1016/j.forsciint.2014.03.024 24763128 10.1016/j.forsciint.2014.03.024

[5] Vass AA (2001) Beyond the grave-understanding human decomposition. Microbiol Today 28:190–193

[6] Pandya JB (2023) General Concept Of Environmental Sciences. Academic Guru Publishing House.

[7] Loy C, Brock F, Dyer C (2023) Investigating diagenesis of archaeological bones from Etton Causewayed enclosure. UK Quaternary Int 660:21–30. 10.1016/j.quaint.2022.12.012

[8] Efremov IA (1940) Taphonomy: a new branch of paleontology. Pan Am Geol 74:81–93

[9] Boaks A, Siwek D, Mortazavi F (2014) The temporal degradation of bone collagen: a histochemical approach. Forensic Sci Int 240:104–110. 10.1016/j.forsciint.2014.04.008 24836839 10.1016/j.forsciint.2014.04.008

[10] Jellinghaus K, Hachmann C, Hoeland K, Bohnert M, Wittwer-Backofen U (2018) Collagen degradation as a possibility to determine the post-mortem interval (PMI) of animal bones: a validation study referring to an original study of Boaks et al.(2014). Int J Leg Med 132:753–763. 10.1007/s00414-017-1747-7 10.1007/s00414-017-1747-7 29177807

[11] Alfsdotter C, Petaros A (2021) Outdoor human decomposition in Sweden: A retrospective quantitative study of forensic-taphonomic changes and postmortem interval in terrestrial and aquatic settings. J Forensic Sci 66:1348–1363. 10.1111/1556-4029.14719 33951184 10.1111/1556-4029.14719

[12] Campobasso CP, Di Vella G, Introna F (2001) Factors affecting decomposition and Diptera colonization. Forensic Sci Int 120:18–27. 10.1016/S0379-0738(01)00411-X 11457604 10.1016/s0379-0738(01)00411-x

[13] Gelderman HT, Boer L, Naujocks T, IJzermans ACM, Duijst WLJM (2018) The development of a post-mortem interval estimation for human remains found on land in the Netherlands. Int J Leg Med 132:863–873. 10.1007/s00414-017-1700-9 10.1007/s00414-017-1700-9 PMC5920129 29110084

[14] Mann RW, Bass WM, Meadows L (1990) Time since death and decomposition of the human body: Variables and observations in case and experimental field studies. J Forensic Sci 35:103–111. 10.1520/JFS12806J 2313251

[15] Sluis I, Duijst W, Krap T (2023) Subaerial Decomposition of Small-Sized Remains in The Netherlands: Important Findings Regarding the PMI of a Four-Year Taphonomic Study. Biology 12(9):1164. 10.3390/biology12091164 37759564 10.3390/biology12091164 PMC10525113

[16] Iqbal MA, Ueland M, Forbes SL (2020) Recent advances in the estimation of post-mortem interval in forensic taphonomy. Aust J Forensic Sci 52(1):107–123. 10.1080/00450618.2018.1459840

[17] Reidsma FH (2022) Laboratory-based experimental research into the effect of diagenesis on heated bone: implications and improved tools for the characterisation of ancient fire. Sci Rep 12(1):17544. 10.1038/s41598-022-21622-5 36323729 10.1038/s41598-022-21622-5 PMC9630262

[18] Delannoy Y, Colard T, Le Garff E, Mesli V, Aubernon C, Penel G, Gosset D (2016) Effects of the environment on bone mass: a human taphonomic study. Leg Med 20:61–67. 10.1016/j.legalmed.2016.04.006 10.1016/j.legalmed.2016.04.006 27161926

[19] Hackett CJ (1981) Microscopical focal destruction (tunnels) in exhumed human bones. Med Sci Law 21(4):243–265. 10.1177/002580248102100403 7321807 10.1177/002580248102100403

[20] Dal Sasso G, Asscher Y, Angelini I, Nodari L, Artioli G (2018) A universal curve of apatite crystallinity for the assessment of bone integrity and preservation. Sci Rep 8(1):12025. 10.1038/s41598-018-30642-z 30104595 10.1038/s41598-018-30642-z PMC6089980

[21] Hedges RE (2002) Bone diagenesis: an overview of processes. Archaeometry 44(3):319–328. 10.1111/1475-4754.00064

[22] Nielsen-Marsh CM, Hedges RE (2000) Patterns of diagenesis in bone I: the effects of site environments. J Archaeol Sci 27(12):1139–1150. 10.1006/jasc.1999.0537

[23] Gupta S, Jawanda MK, Sm M, Bharti A (2014) Qualitative histological evaluation of hard and soft tissue components of human permanent teeth using various decalcifying agents - a comparative study. J Clin Diagn Res. 8(9):ZC69–72. 10.7860/JCDR/2014/10195.4874 10.7860/JCDR/2014/10195.4874 PMC4225979 25386527

[24] Maat GJ, Van Den Bos RP, Aarents MJ (2001) Manual preparation of ground sections for the microscopy of natural bone tissue: update and modification of Frost’s ‘rapid manual method.’ Int J Osteoarchaeol 11(5):366–374. 10.1002/oa.578

[25] Frost HM (1958) Preparation of thin undecalcified bone sections by rapid manual method. Stain Technol 33(6):273–277. 10.3109/10520295809111862 13603078 10.3109/10520295809111862

[26] Schultz M (2001) Paleohistopathology of bone: a new approach to the study of ancient diseases. The Official Publication of the American Association of Physical Anthropologists. Am J Phys Anthropol 116(S33):106–147. 10.1002/ajpa.10024 10.1002/ajpa.10024.abs 11786993

[27] De Boer HH, Aarents MJ, Maat GJR (2012) Staining ground sections of natural dry bone tissue for microscopy. Int J Osteoarchaeol 22(4):379–386. 10.1002/oa.1208

[28] Shehata TP, Krap T (2024) An overview of the heat-induced changes of the chemical composition of bone from fresh to calcined. Int J Leg Med 1–15. 10.1007/s00414-024-03160-z 10.1007/s00414-024-03160-z PMC11004044 38270608

[29] Child AM (1995) Towards and understanding of the microbial decomposition of archaeological bone in the burial environment. J Archaeol Sci 22(2):165–174. 10.1006/jasc.1995.0018

[30] Procopio N, Mein CA, Starace S, Bonicelli A, Williams A (2021) Bone diagenesis in short timescales: insights from an exploratory proteomic analysis. Biology 10(6):460. 10.3390/biology10060460 34071025 10.3390/biology10060460 PMC8224596

[31] Gentile G, Tambuzzi S, Andreola S, Bailo P, Bilato G, Gorini I, Zoja R (2021) Analysis of the corrosive effects of hydrochloric acid (HCl) on human bone: Preliminary microscopic study and observations for forensic purposes. Forensic Sci Int 329. 10.1016/j.forsciint.2021.111095 10.1016/j.forsciint.2021.111095 34775329

[32] Hartnett KM, Fulginiti LC, Di Modica F (2011) The effects of corrosive substances on human bone, teeth, hair, nails, and soft tissue. J Forensic Sci 56(4):954–959. 10.1111/j.1556-4029.2011.01752.x 21447075 10.1111/j.1556-4029.2011.01752.x

[33] Yadav P, Bishariya N, Lather J, Dhattarwal SK, Sharma N, Lohhra A (2024) Assessing the impact of corrosive acids on human bone integrity in forensic context. Forensic Sci Med Pathol. 1–14. https://doi/10.1007/s12024-024-00861-0 10.1007/s12024-024-00861-0 39172313

[34] Williams R, Thompson T, Orr C, Taylor G (2025) Bone Diagenesis and Extremes of Preservation in Forensic Science. Humans 5(1):2. 10.3390/humans5010002

[35] AMBOSS. Pathology techniques (2022) Available online: https://www.amboss.com/us/knowledge/pathology-techniques/ (accessed on 18 December, 2023)

[36] Gurina TS, Simms L (2020) Histology, staining. 32491595

[37] Kim SW, Roh J, Park CS (2016) Immunohistochemistry for pathologists: protocols, pitfalls, and tips. J Pathol Transl Med 50(6):411–418. https://doi/10.4132/jptm.2016.08.08 PMC5122731 27809448

[38] Kenhub (2022) Interpretation of histological sections: stains used in histology. Available online: https://www.kenhub.com/en/library/anatomy/interpretation-of-histologic-sections-stains-used-in-histology (Accessed on 11 January, 2024).

[39] Histology stains (2017) Available online: https://human-embryology.org/wiki/Histology_Stains#Alizarine_Blue (accessed on 16 January, 2024)

[40] Mmegias, Heidenhain Azan Trichrome (2022) Available online: https://mmegias.webs.uvigo.es/02-english/6-tecnicas/protocolos/p-tincion-azan-heidenhain.php (accessed on 16 January, 2024)

[41] StainsFile, Heidenhain’s Azan Trichrome. Available online: https://www.stainsfile.com/protocols/heidenhains-azan-trichrome-for-muscle-and-collagen/ (accessed on 16 January, 2024)

[42] Delannoy Y, Colard T, Cannet C, Mesli V, Hédouin V, Penel G, Ludes B (2018) Characterization of bone diagenesis by histology in forensic contexts: a human taphonomic study. Int J Leg Med 132:219–227. 10.1007/s00414-017-1699-y 10.1007/s00414-017-1699-y 28965197

[43] CliniSciences, Histological Special Stains. Available online: https://www.clinisciences.com/en/buy/cat-histological-special-stains-3926.html# (Accessed on 11 January, 2024).

[44] Histology Research Core, Special stains. Available online: https://histologyresearchcorefacility.web.unc.edu/special-stains/ (Accessed on 10 January, 2024).

[45] Merck, Picrofuchsin stain (2023) Available online: https://www.sigmaaldrich.com/NL/en/product/mm/100199?utm_source=google&utm_medium=cpc&utm_campaign=8693984828&utm_content=119090209864&gclid=EAIaIQobChMI7oGP9MHLhAMV-bCDBx3D0ADLEAAYASAAEgL-SvD_BwE (accessed on 17 January, 2024)

[46] StainsFile, Lillie’s Trichrome. Available online: https://www.stainsfile.com/protocols/lillies-trichrome-for-muscle-and-collagen/ (accessed on 17 January, 2024)

[47] StainsFile, Miller’s Stain. Available online https://www.stainsfile.com/protocols/millers-stain-for-elastic-fibres/ (Accessed on 17 January 2024)

[48] Statlab, Bile Stain Kit (2018) Available online: https://www.statlab.com/pdfs/sds/SSKTHBI.pdf (accessed on 17 January, 2024)

[49] Wisconsin, Histological Staining Techniques. Available online: https://www.vetmed.wisc.edu/lab/histology/histochemical-staining-techniques/ (Accessed on 19 December, 2023).

[50] uOttawa, Histological staining. Avaibleble online https://www.uottawa.ca/research-innovation/histology/services/histological-staining (accessed on 19 December, 2023).

[51] Protocol Online, Histology Staining. Available online: https://www.protocol-online.org/prot/Histology/Staining/ (Accessed on 20 December, 2023).

[52] StainsFile, Hale’s Colloidal Iron . Available online: https://www.stainsfile.com/protocols/hales-colloidal-iron-for-acid-mucosubstances/ (accessed on 18 January, 2024)

[53] DeltaMicrospies, Weigert’s resorcin-fuchsin. Available online: https://www.deltamicroscopies.com/en/products/weigerts-resorcin-fuchsin/ (accessed on 18 January, 2024)

[54] RowleyBio, Fraser-Lendrum Method for Fibrin (2021) Available online: https://rowleybio.com/wp-content/uploads/F-380-Fraser-Lendrum-Method-for-Fibrin.pdf (accessed on 18 January, 2024)

[55] StainsFile, Weigert’s stain. Available online: https://www.stainsfile.com/protocols/weigerts-stain-for-elastic-fibres/ (accessed on 16 January, 2024)

[56] Merck, Pichrofuchsin solution acc. To van Gieson (2006) Available online: https://www.merckmillipore.com/NL/en/product/Picrofuchsin-solution-acc.-to-van-Gieson,MDA_CHEM-100199?ReferrerURL=https%3A%2F%2Fwww.google.com%2F (accessed on 17, January 2024)

[57] Statlab, Methylene Blue Stain Procedure (2019) Available online: https://www.statlab.com/pdfs/ifu/stmbl.pdf (accessed on 19 December, 2023)

[58] Statlab, Methylene Green Stain Procedure (2019) Available online: https://www.statlab.com/pdfs/ifu/stmgp.pdf (accessed on 18 January, 2024)

[59] Bio-Optica, Methyl green pyronin (2012) Available online: https://bio-optica.it/ftp/Sito/technical_datasheet/15003_en.pdf (accessed on 18 January, 2024)

[60] Hashimoto Y, Takano N, Nakamura A, Nakaike S, Yu Z, Endo Y, Arai I (2004) Scratching behavior in NC/Nga mice with dermatitis: Involvement of histamine-induced itching. Allergology Int 53(4):349–358. 10.1111/j.1440-1592.2004.00358.x

[61] DermNet, Histology Stains (2015) Available online: https://dermnetnz.org/topics/histology-stains (Accessed on 19 December, 2023)

[62] MedChemExpress. Crystal Ciolet. Available online: https://www.medchemexpress.com/crystal-violet.html (accessed on 16 January, 2024)

[63] CellaVision, May-Grünwald Giemsa staining. Available online: https://www.cellavision.com/products/reagents/may-grunwald-giemsa-staining (accessed on 17 January, 2024)

[64] Vienna Biocenter. Routine and special histochemical stains. Available online: https://www.viennabiocenter.org/vbcf/histology/routine-and-special-histochemical-stains/ (Accessed on 19 December, 2023)

[65] BioResearch, Malachite Green 1% w/v Aqueous Solution. Available online: https://www.bioresearch.com.jo/ifu/Biological%20Stain/Malakite%20Green.pdf (accessed on 18 January, 2024)

[66] Morphisto, Azocarmine (2024) Available online: https://www.morphisto.de/uploads/tx_aimeos/SDB/MSDS_Azokarmin_10147_EN.pdf (accessed on 27 February, 2024)

[67] Merck, Phloxine B (2017) Available online: https://www.merckmillipore.com/NL/en/product/Picrofuchsin-solution-acc.-to-van-Gieson,MDA_CHEM-100199?ReferrerURL=https%3A%2F%2Fwww.google.com%2F (accessed on 16 January, 2024)

[68] StainsFile, Gridley’s stain. Available online: https://www.stainsfile.com/protocols/gridleys-stain-for-fungi/ (accessed on 18 January, 2024)

[69] Snyder H (2019) Literature review as a research methodology: An overview and guidelines. J Bus Res 104:333–339. 10.1016/j.jbusres.2019.07.039

[70] Jellinghaus K, Urban PK, Hachmann C, Bohnert M, Hotz G, Rosendahl W, Wittwer-Backofen U (2019) Collagen degradation as a possibility to determine the post-mortem interval (PMI) of human bones in a forensic context– A survey. Leg Med 36:96–102. 10.1016/j.legalmed.2018.11.009 10.1016/j.legalmed.2018.11.009 30500672

[71] Bell LS, Skinner MF, Jones SJ (1996) The speed of post mortem change to the human skeleton and its taphonomic significance. Forensic Sci Int 82(2):129–140. 10.1016/0379-0738(96)01984-6 8885373 10.1016/0379-0738(96)01984-6

[72] Jaggers KA, Rogers TL (2009) The effects of soil environment on postmortem interval: a macroscopic analysis. J Forensic Sci 54(6):1217–1222. 10.1111/j.1556-4029.2009.01160.x 19761472 10.1111/j.1556-4029.2009.01160.x

[73] Ramsthaler F, Ebach SC, Birngruber CG, Verhoff MA (2011) Postmortem interval of skeletal remains through the detection of intraosseal hemin traces. A comparison of UV-fluorescence, luminol, Hexagon-OBTI®, and Combur® tests. Forensic Sci. Int. 209(1–3):59–63. 10.1016/j.forsciint.2010.12.011 10.1016/j.forsciint.2010.12.011 21237592

[74] Hoke N, Grigat A, Grupe G, Harbeck M (2013) Reconsideration of bone postmortem interval estimation by UV-induced autofluorescence. Forensic Sci Int 228(1–3):176-e1. 10.1016/j.forsciint.2013.03.013 10.1016/j.forsciint.2013.03.013 23562146

[75] Sterzik V, Jung T, Jellinghaus K, Bohnert M (2016) Estimating the postmortem interval of human skeletal remains by analyzing their optical behavior. Int J Leg Med 130:1557–1566. 10.1007/s00414-016-1395-3 10.1007/s00414-016-1395-3 27262481

[76] Sterzik V, Holz F, Ohlwärther TEN, Thali M, Birngruber CG (2018) Estimating the postmortem interval of human skeletal remains by analyzing their fluorescence at 365 and 490 nm. Int J Leg Med 132:933–938. 10.1007/s00414-017-1759-3 10.1007/s00414-017-1759-3 29256137

[77] Caruso V, Marinoni N, Diella V, Possenti E, Mancini L, Cantaluppi M, Pavese A (2021) Diagenesis of juvenile skeletal remains: A multimodal and multiscale approach to examine the post-mortem decay of children's bones. J. Archaeol. Sci. 135:105477. 10.1016/j.jas.2021.105477

[78] Omar S (2017) *Varkenspoten en -oren: nieuwe inzichten in de schatting van het PMI?* Unpublished bachelor’s thesis, Erasmushogeschool Brussel

[79] Schotsmans EM, Stuart BH, Stewart TJ, Thomas PS, Miszkiewicz JJ (2024) Unravelling taphono-myths. First large-scale study of histotaphonomic changes and diagenesis in bone from modern surface depositions. PloS one, 19(9):e0308440. 10.1371/journal.pone.0308440 10.1371/journal.pone.0308440 PMC11426454 39325784

[80] Singer M (1952) Factors which control the staining of tissue sections with acid and basic dyes. Int. Rev. Cytol. (Vol. 1, pp. 211–255). Academic Press. 10.1016/S0074-7696(08)60012-1

[81] Zinck N, Franz-Odendaal TA (2021) Accurate whole-mount bone and cartilage staining requires acid-free conditions. Anat Rec 304(5):958–960. 10.1002/ar.24526 10.1002/ar.24526 33026708

[82] Sangeetha R, Uma K, Chandavarkar V (2013) Comparison of routine decalcification methods with microwave decalcification of bone and teeth. J Oral Maxillofac Pathol 17(3):386–391. 10.4103/0973-029X.125204 24574657 10.4103/0973-029X.125204 PMC3927340

[83] Daubert v (1993) Merrell Dow Pharmaceuticals Inc, 509 U.S. 579

[84] *Frye v. United States*, 293 F. 1013 (D.C. Cir. 1923)

[85] Gelderman T, Stigter E, Krap T, Amendt J, Duijst W (2021) The time of death in Dutch court; using the Daubert criteria to evaluate methods to estimate the PMI used in court. Leg Med 53. 10.1016/j.legalmed.2021.101970 10.1016/j.legalmed.2021.101970 34601451

[86] National Research Council, Division on Engineering, Physical Sciences, Committee on Applied, Theoretical Statistics, Policy, Committee on Identifying the Needs of the Forensic Sciences Community. (2009) Strengthening forensic science in the United States: a path forward. National Academies Press.

[87] Pachitariu M, & Stringer C (2022) Cellpose 2.0: how to train your own model. Nature methods 19(12):1634–1641. 10.1038/s41592-022-01663-4 10.1038/s41592-022-01663-4 PMC9718665 36344832

[88] van Eekelen L, Litjens G, Hebeda KM (2024) Artificial intelligence in bone marrow histological diagnostics: potential applications and challenges. Pathobiol 91(1):8–17. 10.1159/000529701 10.1159/000529701 PMC10937040 36791682

