# Supplementary material for: Bone histology for forensic anthropology: a technical review on the advances in microstructural analysis of taphonomically altered buried or subaerially exposed bone
Source: Int J Legal Med. 2025 Jul 10;139(6):2921–38. doi: 10.1007/s00414-025-03536-9 (PMC12532741; doi:10.1007/s00414-025-03536-9)
Supplement: Supplementary file 1 — (DOCX 53.0 KB) [file 414_2025_3536_MOESM1_ESM.docx]

**Supplementary Materials**

**Bone histology for forensic anthropology: a technical review on the advances in microstructural analysis of taphonomically altered buried or subaerially exposed bone**

Iris Sluis^1^, Wilma Duijst^1^ and Tristan Krap^1^

^1^Faculty of Law and Criminology, Maastricht University, Minderbroedersberg 4-6, 6211 LK Maastricht, The Netherlands.

1. **Overview histologocial stainings**

The other stains identified during the search that might potentially be suitable for staining bone, but were not effective for staining the organic components, including collagen and osteocytes.

Table 1 Overview of different staining techniques. The table shows the staining, staining colour, application, and the sources.

| Staining | Staining colour | Application | Sources |
| --- | --- | --- | --- |
| Ziehl-Neelsen stain (acid-fast stain) | Red  High mycolic acid content | Acid-fast rods (e.g. Mycobacteria, Nocardia), Protozoa (e.g. oocysts of Cryptosporidium) | [1] |
| Auramine-rhodamine stain | Reddish-yellow  High mycolic acid content | Acid-fast rods (e.g. Mycobacteria, Nocardia), | [1] |
| Acid fast Kinyoun stain | Red  Acid-fast cells  Blue  Non-acid fast cells. | To identify acid-fact bacteria in cells | [2] |
| Silver stain | Black  Certain protein function groups | Various bacteria (e.g., Pseudomonas, Leptospira, Bartonella, Legionella, H. pylori, Treponema), Various fungi (e.g., Aspergillus, Pneumocystis, Coccidioides, Cryptococcus, Candida) | [1, 3] |
| Mucicarmine stain | Bright red  Thick cell walls containing polysaccharides, mucin | Cryptococcus neoformans | [1, 3-4] |
| Sudan Black | Dark black/brown  Lipid-containing structures like triglycerides and lipoproteins | Diagnose atherosclerosis and autosomal dominant leukodystrophy | [3] |
| Oil Red O | Red  Hydrophobic fat or lipids, substances that are traditionally difficult to stain | visualizing atherosclerotic plaques and hepatic and muscular lipid accumulation | [3] |
| Nile red | Fluoresces red  Intracellular lipid droplets | Cell biology, lipid metabolism and other processes involving lipids | [5] |
| Nissl Stain/Cresyl Violet Stain | Blueish/purple  Neural structure in the brain and spinal cord | Studying neuronal morphology and assessing density and distribution of neurons in different brain regions | [3] |
| Indian ink | Halo against dark background  Negative stain with carbon  polysaccharide capsule is not penetrated by ink and appears as a halo around the organism against a dark background | Identify organisms with a polysaccharide capsule such as Cryptococcus neoformans | [6] |
| Thionine staining | Blue violet  Acidic proteins and nucleic acids | Staining of brain sections and visualizing macroscopic lesions | [7-8] |

Table 1 (Continued) Overview of different staining techniques. The table shows the staining, staining colour, application, and the sources.

| Staining | Staining colour | Application | Sources |
| --- | --- | --- | --- |
| Wright-Giemsa | - Neutrophils have purple nuclei, light pink cytoplasm, and reddish-purple granules - Basophils have dark blue or purple nuclei, and very dark purple granules - Eosinophils have blue nuclei, light pink cytoplasm, and red granules - Red blood cells become red or pink - Platelets appear purple | Used to stain peripheral blood smear and bone marrow smears | [9-10] K |
| Alcian Blue | Blue  Mucin stain | To stain acidic polysaccharides such as glycosaminoglycan’s in cartilage and other body structures | [11-12] |
| Fite’s acid fast stain | Red  Acid-fast bacilli  Blue  Background | Used to diagnose leprosy and other bacterial infections such as Nocardiosis | [2] |
| Orcein stain | Dark brown-purple  Inclusion bodies and proteins associated with copper | Identify the inclusion bodies of viruses, commonly used to diagnose hepatitis B | [9] |
| Bismarck Brown | Yellow  Acid mucins | Demonstration of mast cells in peripheral tissues | [13] |
| Alizarin Red | Bright red  Insoluble calcium cations  Dark red  Other metals such as barium, aluminium, mercury and magnesium | Useful for identifying bone or other high calcium structures | [14] |
| Neutral Red | Fluorescent red  Lysosomes | Stain Lysosomes in viable cells | [15] |
| Basement Membrane, Jones | Black  Basement membranes, oxidisable carbohydrates  Background as counterstained | Connective tissue | [16-17] |
| Grocott’s Methenamine Silver (GMS) | Black  Oxidisale carbohydrates, including glycogen and fungi  Background as counterstained. | Fungus | [17-18] |
| Reticulin | Black  Reticulin fibers (collagen type III)  Grey of pink  Background | Connective tissue | [9,17] |
| Luxol Fast Blue | Greenish blue  Myelin  Pink to violet  Cells | White and grey matter, mark myelin within a cell | [17,19] |
| Reticulin stain | Blue/black  Reticular fibres | To demonstrate reticular (retic) fibers | [11-12] |
| Benzidine staining | Dark brown  haemoglobin of erythrocytes | Stain haemoglobin of erythrocytes | [12] |
| Feulgen stain | Red-Rose  DNA | Visualize nuclear chromoatin and to perform semiquantitative assessment of DNA | [12, 20] |

Table 1 (Continued) Overview of different staining techniques. The table shows the staining, staining colour, application, and the sources.

| Staining | Staining colour | Application | Sources |
| --- | --- | --- | --- |
| Aldehyde fuchsin | Blueish-purple  Elastic tissue fibers, beta cell granules, and sulphated mucins | Used for beta cells in pancreas | [9, 11] |
| Alkaline phosphatase | Red/blue  Appositive colonies appear either blue or red to purple, while the surrounding feeder cells appear colorless. | Used for endothelial tissue | [11] |
| Bielshowsky stain | Black  Axons, plaque neurites and tangles  Yellow to brown  Background  Generally brown to dark brown  Plaque and vascular amyloid | Used for neural plaques and tangles | [11, 17] |
| Fontana-Masson | Black/pink or red  Melanin | Can be used for the differentiation of brown pigments (lipofuschine, hemosiderin, melanin), for the deposition of pigment due to treatments like Minocycline type II or for the differential diagnosis of hypomelanosis | [11, 20] |
| Luna stain | Purple/black  Mast cells and elastin | Indicate elastic fibers | [11] |
| Alcian Blue-PAS stain | Rose to red  Glycogen  Blue  Mucosubstances | Highlight glycogen and goblet cells within a tissue section | [19, 21] |
| Bodian’s stain | Black  Nerve fibers | For demonstrating nerve fibers | [21-22] |

1. **Staining protocols**
   1. **Staining protocols undecalcified, embedded bone sections**

Eight of the 45 available staining protocols for undecalcified, embedded bone sections.

- - 1. **Hematoxylin-Eosin**

**Protocol 1: [23]**

1. Stain with Haematoxyilin 5-10 minutes
2. Wash well with Scott s tap water
3. Stain with Eosin 5 minutes
4. Wash in tap water
5. Clear in xylene
6. Mount

**Protocol 2: [24]**

1. Deplasticized slides are stained for 10 min in filtered Harris formula hematoxylin (Fisher #245-651).
2. Slides are rinsed with running tap water until it runs clear (5 min).
3. Sections are differentiated in acidic alcohol (70% EtOH with 1% acetic acid) for 5 s.
4. Slides are rinsed with running tap water for an additional 5 min.
5. Slides are blued in weak ammonia solution (10 mL ammonium hydroxide in 1000 mL of dH2O) for 30s.
6. Slides are washed for an additional 5 min in running tap water.
7. Slides are transferred to 80% EtOH and partially dehydrated for 1 min.
8. They are stained with alcoholic eosin Y (Epredia #71211) for 2 min.
9. Excess eosin is washed away with sequential washes in 100% EtOH. The first is for 1 min, the second for 3 min and the third for 5 min.
10. Sections are then fully dehydrated with three exchanges of xylene (Fisher #016371).
11. Sections are mounted with Epredia media #4112.
    - 1. **Sirius Red/Fast Green [25-28]**
12. Prepare paraffin-embedded tissue sections (approximately 30-50 mm2 , 10-20 μm thick).
13. Deparaffinize the tissue sections with the following steps below:
     1. Xylene, 10 minutes
     2. Xylene 1:1 with 100% ethanol, 10 minutes
     3. 100% ethanol, 10 minutes
     4. 50% ethanol/distilled water, 5 minutes
     5. Distilled water, 5 minutes
14. Transfer individual slides to petri dishes.
15. Load 0.2 - 0.3 ml Dye Solution on each sample, enough to completely immerse the tissue section, and incubate at room temperature for 30 minutes. NOTE: To avoid evaporation of the Dye Solution, place a piece of wet filter paper beneath the slide and cover the petri dish with a lid.
16. Carefully aspirate the Dye Solution.
17. Rinse the stained tissue section with 0.5 ml of distilled water repeatedly until the water runs clear. OPTION: These samples may be observed under a microscope without the following extraction step. Dehydrate in 100% ethanol, followed by a xylene wash, and mount in a resinous medium.
18. Load 1 ml of Dye Extraction Buffer on each sample and gently mix by pipetting until the color is eluted from the tissue section.
19. Collect the eluted Dye Solution and read the OD values at 540 nm and 605 nm with a spectrophotometer.
    - 1. **Methyl Green Pyronin**

**Protocol 1: [29]**

1. Sections on slides are hydrated and immersed in the staining solution for three to five minutes.
2. The sections are rinsed in distilled water for a few seconds and then are blotted with several thicknesses of smooth filter paper. Before the sections have completely dried, they are immersed in the tertiary butyl alcohol mixture for at least two minutes with occasional agitation.
3. The slides are passed through two five-minute changes of xylene and mounted in one of the artificial resins such as Permount or Clarite.

**Protocol 2:[30]**

This stain was used according protocol 1 [Taft (1951)] with a 10 min staining time.

- - 1. **Toluidine Blue**

**Protocol 1: [31]**

1. 5 minutes in 0.1% formic acid
2. Quick rinse in dH_2_O
3. 15 minutes in 70% ethanol
4. 5 minutes Toluidine blue solution
5. Quick rinse in dH_2_O
6. 30 seconds in 70% ethanol (differentiation)
7. 30 seconds in 95% ethanol
8. 30 seconds in 100% ethanol
9. 30 seconds in 100% ethanol
10. Mount the slices

**Protocol 2: [24]**

1. Citric acid buffer was prepared by combining 1.58g citric acid (Fisher #A104), 0.75g of disodium phosphate (JT Baker #3828-01), 1000 mL dH2O, and adjusting the pH to 3.7.
2. 2 g of Toluidine blue O (Fisher #T161) were added to 100 mL of buffer, mixed thoroughly, and then filtered. The pH was checked to be sure it remained at pH 3.7.
3. Deplasticized and rehydrated slides were immersed in stain for 15–20 min at room temperature.
4. The slides were rinsed in three changes of citrate buffer and blotted dry.
5. Slides were allowed to fully dry on the benchtop for at least 20 min.
6. Sections were dehydrated rapidly through two changes of tert-butanol (Fisher #A401), one change of 50%/50% tert-butanol/toluene, and two final changes of toluene (Fisher #T324).
7. Slides were mounted with Epredia media #4112.
   - 1. **Masson-Goldner staining**

**Protocol 1: [32]**

**For Staining Methyl Methacrylate Sections**

1. Flatten sections with a drop of distilled water.
2. Stain 7 min with 50:50 Harris' hematoxy1in:ferric chloride (made fresh with each staining procedure).
3. Rinse two times with warm tap water.
4. Stain 5 min with Ponceau de xylidineacid fuchsin staining solution.
5. Rinse with 1% acetic acid.
6. Stain 6 min with 1% phosphomolybdic acid.
7. Rinse with 1% acetic acid.
8. Stain 2 min with light green staining solution.
9. Rinse with 1% acetic acid.
10. Dry on bibulous paper overnight.
11. Rinse in xylene with 2% ethanol to clear and soften.
12. Mount and coverslip

**For Staining Glycol Methacrylate Sections**

1. Wet slides with d HzO; shake off excess.
2. Stain 3 min with 50:50 Harris' hematoxy1in:ferric chloride (made fresh with each staining procedure).
3. Rinse two times with warm tap water.
4. Stain 12 min with Ponceau de xylidine-acid fuchsin staining solution.
5. Rinse with 0.5% acetic acid.
6. Stain 3 min with 0.5% phosphomolybdic acid (0.5 g phosphomolybdic acid, 100 ml d HzO).
7. Rinse with 0.5% acetic acid.
8. Stain 5 min with light green.
9. Rinse quickly in 0.5% acetic acid.
10. Air dry.
11. Dip in xylene to clear and coverslip.

**For Staining Thick Sections of Spurr Resin**

1. Wet sections with d H20; shake off excess.
2. Stain 3 min with ferric chloride with heat.
3. Rinse quickly with warm tap water and dry with heat.
4. Stain 25 sec with hematoxylin with heat.
5. Rinse quickly with warm tap water and dry with heat.
6. Stain 8 min with Ponceau de xylidineacid fuchsin solution with heat.
7. Rinse quickly with 0.5% acetic acid.
8. Stain 3 min with 0.5% phosphomolybdic acid with heat.
9. Rinse quickly with 0.5% acetic acid. heat.
10. Stain 3-5 min with light green with
11. Rinse quickly with 0.5% acetic acid.
12. Air dry and coverslip.

**Protocol 2: [23]**

1. Wash with alkaline alcohol (90mls of 80% ethanol and 10mls of 25% ammonia for 20 minutes)
2. Rinse in water
3. Rinse in distilled water
4. Stain with Weigert s Haematoxylin for 10 minutes
5. Rinse in distilled water
6. Stain with Ponceau-Fuchsin final solution 5 minutes
7. Rinse with 1% acetic acid 15 seconds
8. Stain in phosphomolybdic acid-orange G solution 5 minutes
9. Rinse with 1% acetic acid 15 seconds
10. Stain with light green 5 minutes
11. Rinse with 1% acetic acid 3 changes
12. Rinse in distilled water
13. Mount

**Protocol 3: [33]** Thin plastic sections

1. Slides must be deplastified using XYLENE ONLY (see Note 25); acetone and MMA weaken the contrast between osteoid and mineralized bone. Always check stains for mold growth before proceeding, and, if present, prepare new Goldner’s modified trichrome solutions.
2. Remove plastic film.
3. Place slides in warm (40–60 °C) xylene (discard xylene after use) for 40 min.
4. Place slides in cool (room temperature) xylene with agitation (shaker table, 1 Hz; discard after use) for 20 min.
5. Repeat step 3 (discard xylene after use) for 20 min.
6. Rehydrate through graded series of EtOH (can be done on linear stainer): 100 % EtOH for 2–5 min, 100 % EtOH for 2–5 min, 95 % EtOH for 2–5 min, 70–80 % EtOH for 2–5 min, and DI H2O for 2–5 min.
7. Stain in working solution of Weigert’s iron hematoxylin for 15 min.
8. Rinse (dip) in DI H2O.
9. Wash in gently running tap (basic pH) water to blue hematoxylin. The initial wine-colored histological sections should transition to blue/black color in approximately 15 min.
10. Rinse (dip) in DI H2O.
11. Stain in ponceau–acid fuchsin for 15 min.
12. Rinse (dip and shake) in 1 % acetic acid. Repeat.
13. Quickly dip in DI H2O to remove acid.
14. Stain in phosphomolybdic acid–Orange G for 8 min.
15. Rinse (dip and shake) in 1 % acetic acid. Repeat.
16. Quickly dip in DI H2O to remove acid.
17. Stain in Light Green SF Yellowish for 15 min.
18. Rinse (dip and shake) in 1 % acetic acid. Repeat.
19. Quickly dip in DI H2O to remove acid (NOTE: over-rinsing will remove staining).
20. Dehydrate, clear, and mount (can be done on linear stainer) through following graded ethanol immersions: 70–80 % EtOH for 2–5 min, 95 % EtOH for 2–5 min, 100 % EtOH for 2–5 min, and 100 % EtOH for 2–5 min.
21. Clear in xylene for 2–5 min.
22. Coverslip with quick-hardening mounting medium.

**Protocol 4: [33]** Thick plastic sections

1. Place each section in a labeled plastic cassette.
2. Immerse cassettes in DI H2O for 5–10 min.
3. Briefly sonicate cassettes to remove bone dust using ultrasound bath.
4. Immerse cassettes in fresh DI H2O until ready to proceed.
5. Stain in working solution of Weigert’s iron hematoxylin for 15 min.
6. Wash in gently running tap (basic pH) water to blue hematoxylin. The initial wine-colored histological sections should transition to blue/black color in 15 min.
7. Sonicate cassettes/sections in DI H2O to remove precipitate.
8. Rinse in DI H2O.
9. Stain in ponceau–acid fuchsin for 15 min. At this point, a second run can be started in the Weigert’s hematoxylin.
10. Pour off stain (save), and quickly rinse in 1 % acetic acid (i.e., quickly cover cassettes with acid solution, then immediately pour off). Repeat.
11. Rinse slides quickly in DI H2O.
12. Blot cassettes with paper towels, bibulous paper, or Kimwipes to remove excess water.
13. Stain in phosphomolybdic acid–Orange G for 8 min.
14. Pour off stain (save), and quickly rinse in 1 % acetic acid (i.e., quickly cover cassettes with acid solution, then immediately pour off). Repeat.
15. Rinse quickly in DI H2O.
16. Blot cassettes with paper towels, bibulous paper, or Kimwipes to remove excess water.
17. Stain in Light Green SF Yellowish for 15 min.
18. Pour off stain (save), and quickly rinse in 1 % acetic acid (i.e., quickly cover cassettes with acid solution, then immediately pour off). Repeat.
19. Cover cassettes with DI H2O and drain.
20. Individually dehydrate sections by carefully removing section from cassette. Using forceps, immerse section in 70 % EtOH for ten dips, 95 % EtOH for ten dips, 100 % EtOH for ten dips, and 100 % EtOH for ten dips.
21. Allow the specimen to air-dry and then clamp between two glass slides overnight. This will flatten the sections and promote ease in cover-slipping. Smaller specimens may have to be clamped before they are fully dry due to their extreme warping of the plastic. Use your finger to flatten the specimen before applying the clamping slide.
22. Immerse flattened sections in xylene to clear and coverslip with quick-hardening mounting medium.

**Protocol 5: [34]**

1. Deparaffinization of the sections
2. Hansen's hematoxylin 1 - 5 minutes
3. Washing with acidified distilled water 5 minutes
4. Masson's Ponceau-Fuchsin solution 5 minutes or more
5. Washing with acidulated distilled water 5 minutes
6. Orange G - phosphotungstic acid (PTA) 15 seconds - 30 minutes
7. Washing with acidified distilled water 5 minutes
8. Masson's light green solution 5 minutes
9. Washing with acidulated distilled water 5 minutes
10. Dehydration in growing alcohols
11. Rinsing in xylol
12. Mounting in neutral medium

**Protocol 6: [24]**

1. Deplasticized sections were first treated with Bouin's solution (MilliporeSigma #HT10132) overnight at ambient temperature.
2. Slides were then washed with running tap water for 10 min until excess color was washed away.
3. The sections were then incubated with Weigert's hematoxylin (mixed the same as above in Safranin O) for 10 min.
4. The samples were again rinsed with running tap water for 10 min.
5. Slides were next stained with Ponceau Acid Fuchsin (Electron Microscopy Sciences #26386-04) for 5 min.
6. They were then quickly rinsed in 1% acetic acid solution (see Safranin O above).
7. Then, they were transferred to Phosphomolybdic Acid-Orange G (Electron Microscopy Sciences #26386-06) solution for 10 min to decolorize the collagen.
8. The slides were quickly rinsed again in 1% acetic acid.
9. They were then stained with Light Green (Electron Microscopy Sciences #26386-07) for 5 min.
10. A final incubation in 1% acetic acid for five min happens next.
11. The sections are then blotted (but not dried) to remove excess liquid.
12. Dehydration is completed through 5 min incubations in 95% EtOH (three times), 100% EtOH (three times), and xylene (three times).
13. The sections are mounted with Epredia media #4112.
    - 1. **Periodic acid-Schiff reaction (PAS reaction) [30]**
14. 0.5% periodic acid 10 min
15. Wash with SO2 water 5 min
16. Schiffs reagent 30 min
17. Wash in water and air dry
    - 1. **Von Kossa staining**

**Protocol 1: [23]**

1. Place in silver nitrate solution and expose to strong light until mineralised bone turns black (approx 10 mins)
2. Wash in distilled water three times
3. Threat with sodium thiosulfate for 5 minutes
4. Wash in distilled water
5. Counterstain with Safrinin O
6. Clear in xylene
7. Mount

**Protocol 2: [33]** Von kossa with MacNeal’s Tetrachrome Counterstain

1. Deplastify sections in either acetones (preferred) or MMA+ xylene (osteoid is slightly bluer with acetone).
2. Hydrate sections through 100 % EtOH, 95 % EtOH, 70 % EtOH, and DI H2O. Repeat.
3. Stain in silver nitrate solution in the dark for 10 min.
4. Rinse in DI H2O three times for 1 min each.
5. Stain in sodium carbonate–formaldehyde solution for 2 min.
6. Rinse in DI H2O for 1 min. Repeat. Add potassium ferricyanide to Farmer’s diminisher.
7. Stain in Farmer’s diminisher (time is critical) for 30 s.
8. Wash in running tap water for 20 min.
9. Rinse in DI H2O for 1 min.
10. Stain in MacNeal’s tetrachrome solution for 20 min.
11. Rinse in DI H2O for each specimen three times for 1 min each. Do not let sit in water after rinses.
12. Dehydrate in one change of 70 % EtOH, 95 % EtOH, and 100 % EtOH.
13. Clear in two changes of xylene for 5 min. Repeat.
14. Coverslip with xylene-based mounting media (Eukitt)

**Protocol 3: [33]** Von Kossa Stain with Nuclear Fast Red Counterstain

1. Deplastify slides by placing slides in 100 % acetone solution for 20 min. Remove slide and replace in second 100 % acetone solution for 20 min. Remove slide and place in third 100 % acetone solution for 20 min.
2. Hydrate slides in DI H2O.
3. Place slides in 5 % silver nitrate solution for 1 h.
4. Rinse, with agitation, in DI H2O repeating three times.
5. Place slides in photographic developer solution for 2 min and rinse in DI H2O. Repeat. 6. Place in sodium thiosulfate solution for 5 min.
6. Wash in running tap water for 2 min.
7. Counterstain in nuclear fast red solution for 5 min.
8. Wash thoroughly in running tap water for 2 min.
9. Dehydrate and clear through 95 % EtOH, 100 % EtOH, and xylene for 2 min. Repeat.
10. Mount with resinous medium

**Protocol 4: [24]**

1. A 5% solution of silver nitrate (Fisher #S486) is made and filtered.
2. Deplasticized slides are incubated in the silver nitrate solution at room temperature for 30 min in the dark.
3. The slides are then washed with three changes of dH2O for 5 min each.
4. 5% sodium carbonate- formaldehyde solution is prepared as follows: 75 mL of dH2O and 25 mL 37% formaldehyde (Fisher #33314) are combined, then 5 g of sodium carbonate (Sigma #S-7795) was dissolved in the mixture.
5. Slides are differentiated in the sodium carbonate-formaldehyde solution for 2 min.
6. The slides are then washed for 10 min in running tap water.
7. Counterstaining is achieved with methyl green pyronin (Sigma #HT70116) for 20 min.
8. The slides are washed in two 1 min changes of dH2O.
9. The samples are then dehydrated in on change of 95% EtOH for 1 min, and two changes of 100% EtOH for 1 min each, then cleared through two changes of xylene, (2 min each).
10. Coverslips are applied using Epredia media #4112.
    - 1. **Safranin O**

**Protocol 1: [24]**

1. Deplasticized slides are placed in Weigert’s solution for 7 min to stain nuclei. Weigert’s is made by mixing equal parts Weigert Solution A (Electron Microscopy Sciences #26044-06) and B (Electron Microscopy Sciences #26044-16).
2. Slides are washed for 10 min with running tap water to remove excess stain.
3. The sections are then incubated in 0.01% Fast Green solution (0.1 g Fast Green, FCF (Fisher #BP123 in 1000 mL dH2 O) for 5 min.
4. Samples are quickly dipped in 1% acetic acid (10 mL glacial acetic acid in 1000 mL dH2 O).
5. The slides are then stained with 0.1% Safranin O for 5 min (0.1 g Safranin O, MilliporeSigma #S-8884 in 1000 mL dH2 O).
6. The slides are rinsed quickly in dH2 O.
7. They are then incubated in 1% acetic acid for 5 min.
8. The sections are next sequentially passed through sequential washes (2 min each) of 95% ethanol (twice), 100% EtOH (twice), xylene (3 times).
9. Mounting was accomplished with Epredia media #4112.

**Protocol 2: [24]**

1. Deplasticized slides are placed in Weigert's solution for 7 min to stain nuclei. Weigert's is made by mixing equal parts Weigert Solution A (Electron Microscopy Sciences #26044-06) and B (Electron Microscopy Sciences #26044-16).
2. Slides are washed for 10 min with running tap water to remove excess stain.
3. The sections are then incubated in 0.01% Fast Green solution (0.1 g Fast Green, FCF (Fisher #BP123 in 1000 mL dH2O) for 5 min.
4. Samples are quickly dipped in 1% acetic acid (10 mL glacial acetic acid in 1000 mL dH2O).
5. The slides are then stained with 0.1% Safranin O for 5 min (0.1 g Safranin O, MilliporeSigma #S-8884 in 1000 mL dH2O).
6. The slides are rinsed quickly in dH2O.
7. They are then incubated in 1% acetic acid for 5 min.
8. The sections are next sequentially passed through sequential washes (2 min each) of 95% ethanol (twice), 100% EtOH (twice), xylene (3 times).
9. Mounting was accomplished with Epredia media #4112.
   1. **Staining protocols undecalcified, non-embedded bone sections**

Two of the 45 available staining protocols for undecalcified, non-embedded bone sections.

- - 1. **Hematoxylin-Eosin**

**Protocol 1: [35]**

1. Place the section in a perforated holder and rinse overnight with gently running water, avoiding direct water flow on the section.
2. Dilute 100 cm³ of Mayer’s haematoxylin to 25% with demineralized water and filter the solution.
3. Transfer the section to a porcelain staining cup and stain for 4 min in haematoxylin, gently moving the cup for even distribution.
4. Rinse the section under gently running tap water for 10 min, stirring occasionally.
5. Further rinse for 1 min in a container with 400 cm³ demineralized water to remove excess haematoxylin.
6. If needed, filter 400 cm³ eosin solution and stain the section for 90 sec, stirring gently.
7. Rinse the section in a new container with demineralized water for 30 sec while moving the cup.
8. Dehydrate the section by placing it in 100% ethanol for 5 min, then repeat with a fresh container of ethanol.
9. Transfer the section through three containers of xylene, 5 min in each.
10. Mount the section on a glass slide with Aquatex and a cover slip, allowing it to dry horizontally for 30 min.

**Protocol 2: [36]**

1. Place the bone section in a perforated 50 mL Falcon tube.
2. Place the Falcon tube in a 25% hematoxylin solution for 8 minutes. Gently move the tube back and forth a few times to ensure the bone section is evenly exposed to the stain.
3. Transfer to an eosin solution for 8 minutes.
4. 10 minutes in tap water.
5. 1 minute in dH_2_O.
6. 3 minutes in 70% ethanol.
7. 3 minutes in 90% ethanol.
8. 3 minutes in 100% ethanol.
9. Mount the slice.
   - 1. **Sirius Red/Fast Green [36]**
10. Place a wet Torq paper at the bottom of a petri dish (one for each slice to be stained).
11. Transfer the slices to the Petri dishes.
12. Apply 0.2 to 0.3 mL of Dye Solution to each sample, ensuring that each slice is submerged.
13. Place the lid on the dish.
14. Incubate for 30 minutes at room temperature.
15. Carefully pipette away the Dye Solution.
16. Rinse the slice with 0.5 mL of distilled water repeatedly until the water runs clear.
17. Dehydrate in 100% ethanol.
18. Mount the slice.
    1. **Mount the slice [36]**
19. Let the bone section air dry for 2 minutes.
20. Prepare a microscope slide and clean both sides with a bit of alcohol.
21. Using a disposable plastic pipette, add a few drops of mounting medium onto the slide.
22. Remove the bone section from the water/Falcon tube/Petri dish with a soft brush and plastic tweezers, and place the section on top of the mounting medium drops.
23. Gently press both sides of the section on a piece of Torq paper.
24. Immediately add another drop of mounting medium on top of the section, ensuring it remains centered on the slide (adjust with a brush if needed).
25. Carefully place the coverslip on top:
    1. Bring one corner into contact with the mounting medium.
    2. Slide this corner to the desired position.
    3. Lower the other corner of that side of the coverslip.
    4. Gently lower the other side of the coverslip over the section, ensuring no air is trapped underneath.
26. Let it dry for half an hour, lying flat with the section facing upward.
27. Examine under the microscope.

**Literature:**

1. AMBOSS, Pathology techniques, 2022. Available online: <https://www.amboss.com/us/knowledge/pathology-techniques/> (accessed on 18 December, 2023)

2. LeicaBiosystems, Acid Fast Bacteria and Acid Fast Staining, 2022. Available online: <https://www.leicabiosystems.com/knowledge-pathway/acid-fast-bacteria-and-acid-fast-staining/> (accessed on 18 January, 2024)

3. Gurina, T. S., & Simms, L. (2020). Histology, staining.

4. Statlab, Mucicarmine stain (stock) procedure, 2019. Available online: <https://www.statlab.com/pdfs/ifu/stmuc.pdf> (accessed on 18 December, 2023)

5. Greenspan, P., Mayer, E. P., & Fowler, S. D. (1985). Nile red: a selective fluorescent stain for intracellular lipid droplets. J. Cell Biol. 100(3):965-973. https://doi.org/10.1083/jcb.100.3.965

6. AMBOSS, Overview of fungal infections, 2023. Available online: [https://www.amboss.com/us/knowledge/overview-of-fungal-infections#Z2ca39e2e1b4029bcdf9ab1a13d5fcb3f](https://www.amboss.com/us/knowledge/overview-of-fungal-infections#Z2ca39e2e1b4029bcdf9ab1a13d5fcb3f) (accessed on 19 December, 2023)

7. Depts. Washington, Thionin Stain, Modified Wisconsin. [https://depts.washington.edu/rubelab/protocols/Thionin-stain.html#:~:text=Thionin%20stain&text=This%20stain%20is%20specific%20for,of%20the%20final%20staining%20solution](https://depts.washington.edu/rubelab/protocols/Thionin-stain.html#:~:text=Thionin%20stain&text=This%20stain%20is%20specific%20for,of%20the%20final%20staining%20solution) (accessed on 16 January, 2024)

8. MDBiosciences, Histochemical Staining Methods. Available online: <https://www.mdbhistopath.com/histopathology-histochemical-staining> (accessed on 19 December, 2023).

9. Kenhub, Interpretation of histological sections: Stains used in histology, 2022. Available online: <https://www.kenhub.com/en/library/anatomy/interpretation-of-histologic-sections-stains-used-in-histology> (Accessed on 11 January, 2024).

10. uOttawa, Histological staining. Avaibleble online <https://www.uottawa.ca/research-innovation/histology/services/histological-staining> (accessed on 19 December, 2023).

11. DermNet, Histology Stains, 2015. Available online: <https://dermnetnz.org/topics/histology-stains> (Accessed on 19 December, 2023)

12. Vienna Biocenter, Routine and special histochemical stains. Available online: <https://www.viennabiocenter.org/vbcf/histology/routine-and-special-histochemical-stains/> (Accessed on 19 December, 2023)

13 StainsFile , Bismarck Brown Y. Available online: <https://www.stainsfile.com/dyes/bismarck-brown-y/> (accessed on 18 January, 2024)

14. Histology stains, 2017. Available online: [https://human-embryology.org/wiki/Histology_Stains#Alizarine_Blue](https://human-embryology.org/wiki/Histology_Stains#Alizarine_Blue) (accessed on 16 January, 2024)

15. ThermoFisher, Neutral Red, 2020. Available online: <https://www.thermofisher.com/order/catalog/product/N3246> (accessed on 17 January, 2024)

16. StainsFile, Jones’ Impregnation. Available online [https://www.stainsfile.com/protocols/jones-impregnation-for-basement-membranes /](https://www.stainsfile.com/protocols/jones-impregnation-for-basement-membranes%20/) (accessed on 18 January, 2024)

17. Wisconsin, Histological Staining Techniques. Available online: <https://www.vetmed.wisc.edu/lab/histology/histochemical-staining-techniques/> (Accessed on 19 December, 2023).

18. StainsFile, Gomori’s Methanamine Silver. Available online <https://www.stainsfile.com/protocols/gomoris-methenamine-silver-for-glycogen-and-fungi/> (accessed on 17 January, 2024)

19. Histology Research Core, Special stains. Available online: <https://histologyresearchcorefacility.web.unc.edu/special-stains/> (Accessed on 10 January, 2024).

20. CliniSciences, Histological Special Stains. Available online: [https://www.clinisciences.com/en/buy/cat-histological-special-stains-3926.html#](https://www.clinisciences.com/en/buy/cat-histological-special-stains-3926.html) (Accessed on 11 January, 2024).

21. Protocol Online, Histology Staining. Available online: <https://www.protocol-online.org/prot/Histology/Staining/> (Accessed on 20 December, 2023).

22. WebPath, Bodian’s Method. Available online: <https://webpath.med.utah.edu/HISTHTML/MANUALS/BODIAN.PDF> (accessed on 18 January, 2024)

23. Goldschlager, T., Abdelkader, A., Kerr, J., Boundy, I., & Jenkin, G. (2010). Undecalcified bone preparation for histology, histomorphometry and fluorochrome analysis. Journal of visualized experiments: JoVE, (35), 1707. https://doi.org/10.3791/1707

24. Fretz, J. A., & Troiano, N. W. (2024). Optimized Methyl methacrylate embedding of small and large undecalcified bones. MethodsX, 13, 103046. https://doi.org/10.1016/j.mex.2024.103046

25. Boaks, A., Siwek, D. and Mortazavi, F., 2014. The temporal degradation of bone collagen: a histochemical approach. Forensic Sci. Int. 240:104-110. https://doi.org/10.1016/j.forsciint.2014.04.008

26. Chondrex Inc. (2008) A simple quantitative micro-assay kit for collagen and non-collagenous proteins. https://www.chondrex.com/documents/9046.pdf. (Accessed on 31 March, 2025)

27. Jellinghaus, K., Hachmann, C., Hoeland, K., Bohnert, M., & Wittwer-Backofen, U. (2018). Collagen degradation as a possibility to determine the post-mortem interval (PMI) of animal bones: a validation study referring to an original study of Boaks et al.(2014). Int. J. Leg. Med. 132:753-763. https://doi.org/10.1007/s00414-017-1747-7

28. Jellinghaus, K., Urban, P. K., Hachmann, C., Bohnert, M., Hotz, G., Rosendahl, W. and Wittwer-Backofen, U., 2019. Collagen degradation as a possibility to determine the post-mortem interval (PMI) of human bones in a forensic context– A survey. Leg. Med. 36 :96-102. https://doi.org/10.1016/j.legalmed.2018.11.009

29. Taft, E. B. (1951). The problem of a standardized technic for the methyl-green-pyronin stain. Stain Technology, 26(3), 205-212. https://doi.org/10.3109/10520295109113209

30. Franklin, R. M., & Martin, M. T. (1980). Staining and histochemistry of undecalcified bone embedded in a water-miscible plastic. Stain Technology, 55(5), 313-321. https://doi.org/10.3109/10520298009067260

31. Peev, S., Parushev, I., & Yotsova, R. (2024). A Modified Protocol for Staining of Undecalcified Bone Samples Using Toluidine Blue—A Histological Study in Rabbit Models. Applied Sciences, 14(1), 461. https://doi.org/10.3390/app14010461

32. Gruber, H. E. (1992). Adaptations of Goldner's Masson trichrome stain for the study of undecalcified plastic embedded bone. Biotechnic & histochemistry, 67(1), 30-34. https://doi.org/10.3109/10520299209110002

33. Bemenderfer, T. B., Harris, J. S., Condon, K. W., & Kacena, M. A. (2014). Tips and techniques for processing and sectioning undecalcified murine bone specimens. Skeletal Development and Repair: Methods and Protocols, 123-147. https://doi.org/10.1007/978-1-62703-989-5_10

34. Panes, C., Ponce, N., Ottone, N. E., Valdivia-Gandur, I., Beltrán, V., & Vásquez, B. (2024). Modified Goldner Trichrome for Non-decalcified Mineralized Tissue Plastinated and Embedded in Resin. International Journal of Morphology, 42(2). https://doi.org/10.4067/S0717-95022024000200516

35. De Boer, H. H., Aarents, M. J., & Maat, G. J. R. (2012). Staining ground sections of natural dry bone tissue for microscopy. Int. J. Osteoarchaeol. 22(4):379-386. https://doi.org/10.1002/oa.1208

36. Nienkemper, J. (2018). Exploring new methods to determine the PMI of modern remains. (unpublished master thesis). University of Amsterdam.
